# Supplementary material for: Identification of Novel Antifungal Skeleton of Hydroxyethyl Naphthalimides with Synergistic Potential for Chemical and Dynamic Treatments
Source: Molecules. 2022 Dec 2;27(23):8453. doi: 10.3390/molecules27238453 (PMC9739515; doi:10.3390/molecules27238453)
Supplement: Supplementary file 1 [file molecules-27-08453-s001.zip › molecules-2033781-supplementary.pdf]

Article

Identification of Novel Antifungal Skeleton of Hydroxyethyl Naphthalimides with Synergistic Potential for Chemical and Dynamic Treatments

Pengli Zhang [1,2,3], Vijai Kumar Reddy Tangadanchu [4,*] and Chenghe Zhou [1,*]

1 Institute of Bioorganic & Medicinal Chemistry, Key Laboratory of Applied Chemistry of Chongqing Municipality, School of Chemistry and Chemical Engineering, Southwest University, Chongqing 400715, China; zhangpengli@zidd.ac.cn
2 Zhongshan Institute for Drug Discovery, Shanghai Institute of Materia Medica, Chinese Academy of Sciences, Zhongshan 528400, China
3 Drug Discovery and Development Center, Shanghai Institute of Materia Medica, Chinese Academy of Sciences, Shanghai 201203, China
4 Department of Radiology, Washington University School of Medicine in St Louis, St. Louis, MO 63110, USA
* Correspondence: tvijaikumarreddy@wustl.edu (V.K.R.T.); zhouch@swu.edu.cn (C.Z.)

Characterizations of some representative compounds

1.1 Spectra of compound **3a**.

^{1}H NMR spectrum

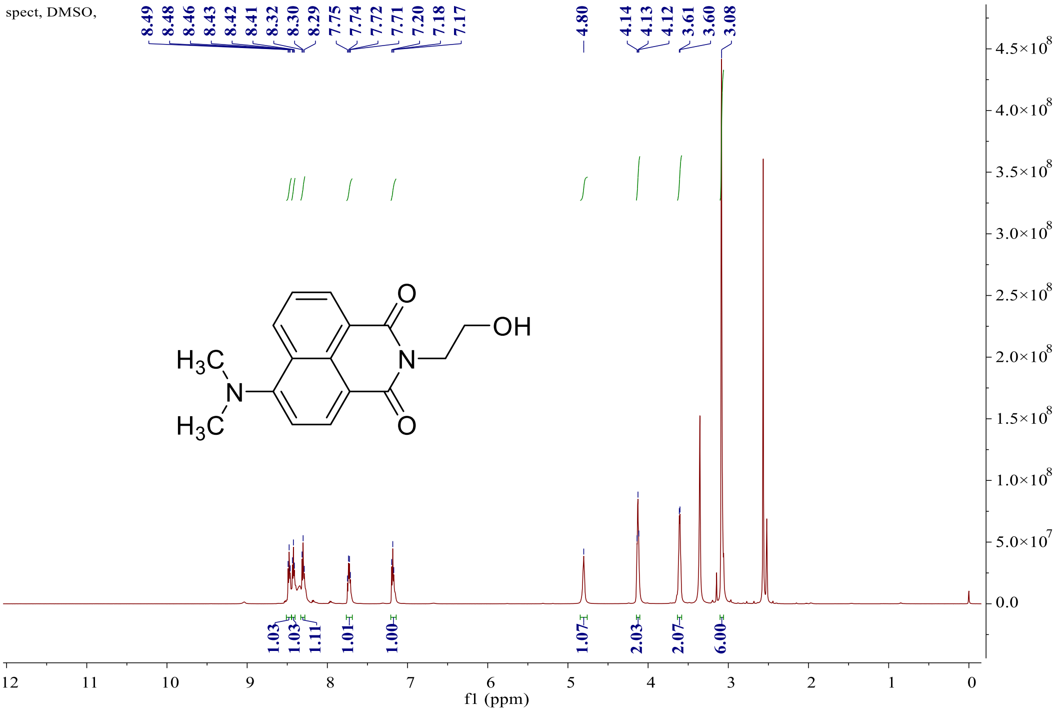

^{13}C NMR spectrum

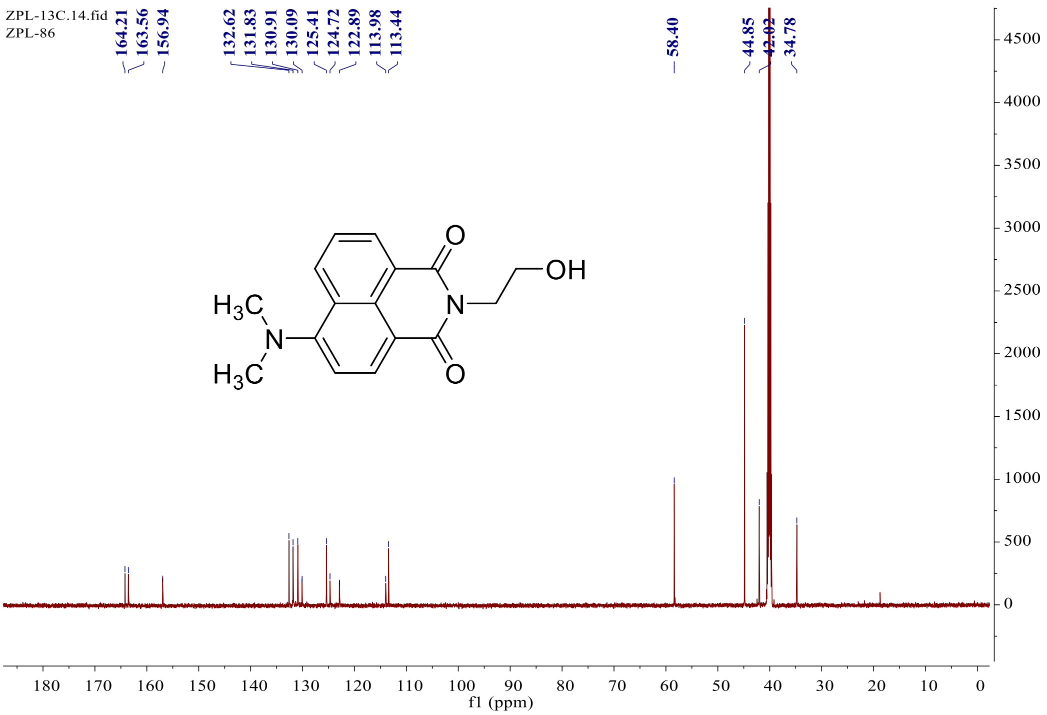

HRMS spectrum

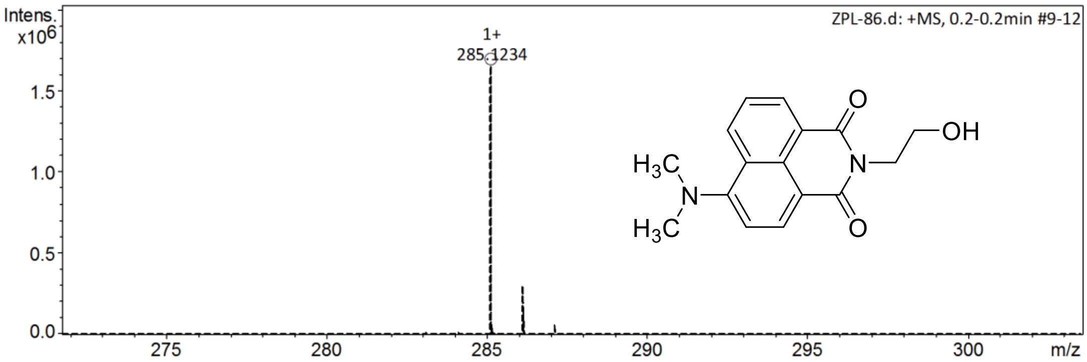

HPLC spectrum

No.	RT	Area (%)	Concentration (%)	BC
1	1. 853	5681	0. 031	BB
2	3. 540	18542525	99. 964	BB
3	5. 020	1006	0. 005	BB
		18549212	100. 000	

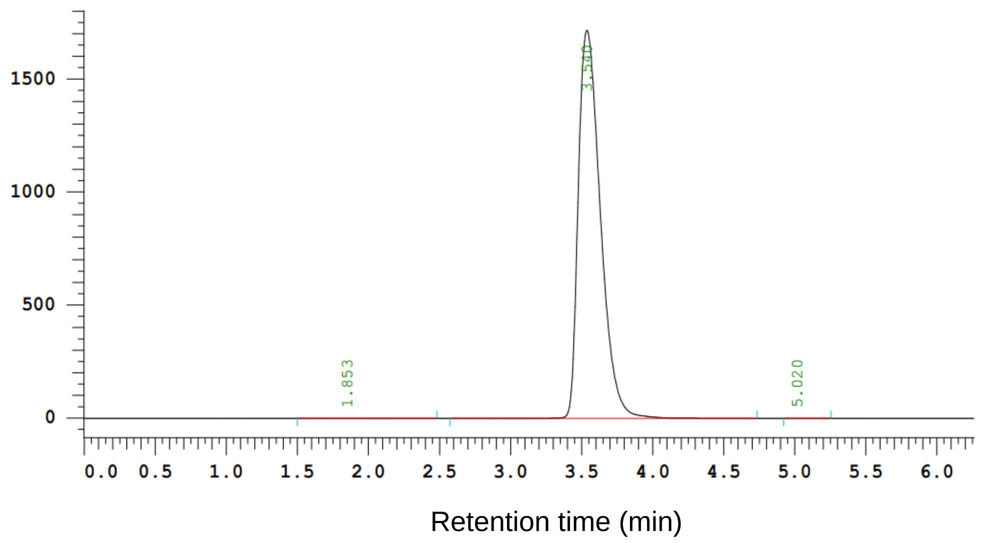

1.2 Spectra of compound **4a**.

^{1}H NMR spectrum

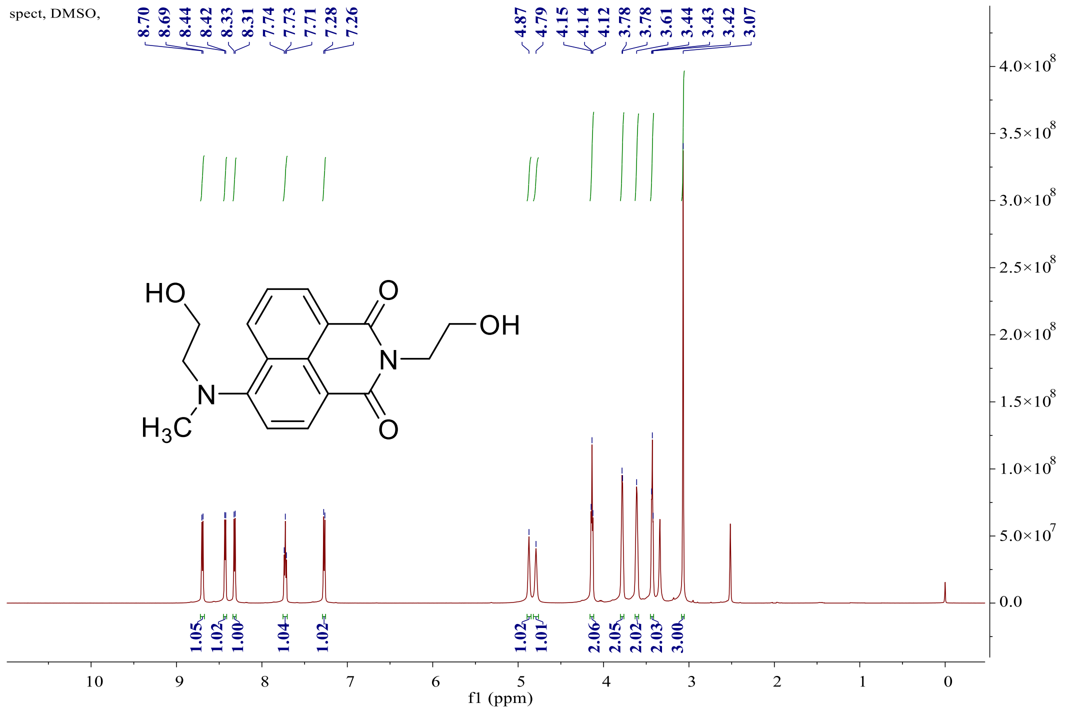

^{13}C NMR spectrum

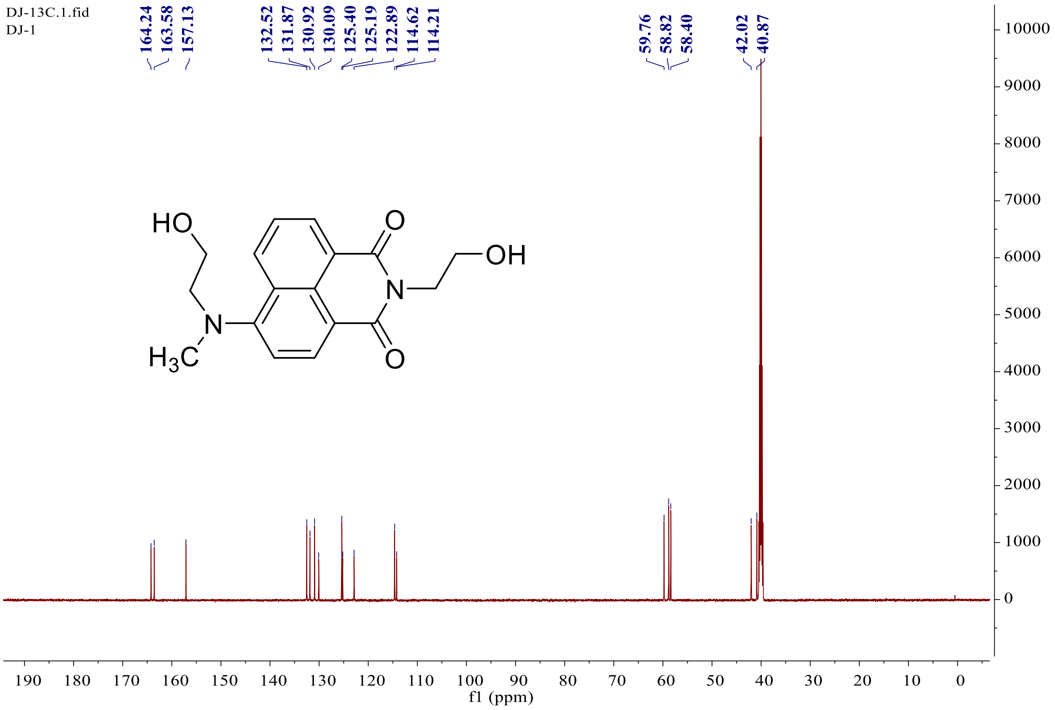

HRMS spectrum

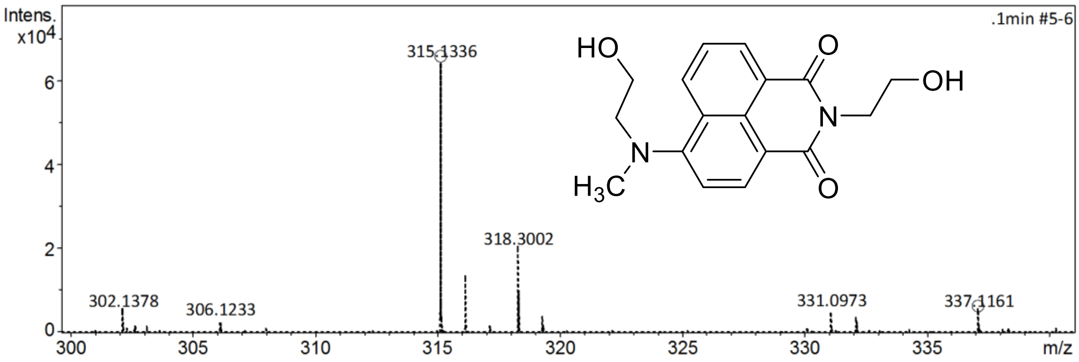

HPLC spectrum

No.	RT	Area (%)	Concentration (%)	BC
1	1. 887	10064	0. 818	BB
2	2. 520	752	0. 061	BB
3	3. 313	1218945	99. 120	BB
		1229761	100. 000	

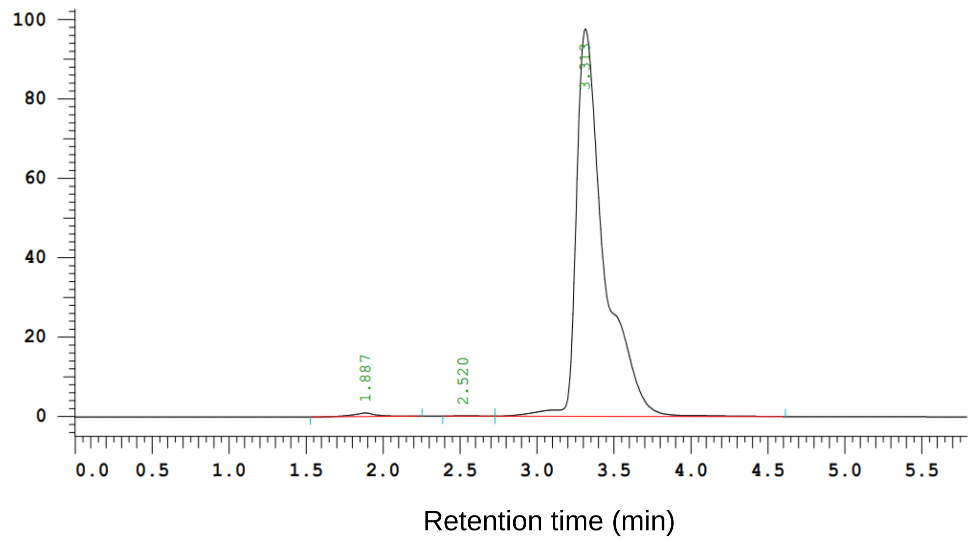

1.3 Spectra of compound **4b**.

HRMS spectrum

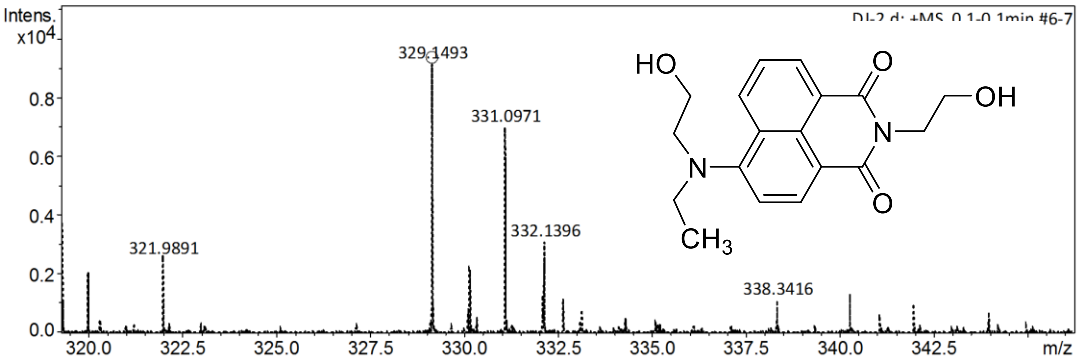

HPLC spectrum

No.	RT	Area (%)	Concentration (%)	BC
1	1. 840	7864	0. 660	BB
2	3. 287	1183101	99. 340	BB
		1190965	100. 000	

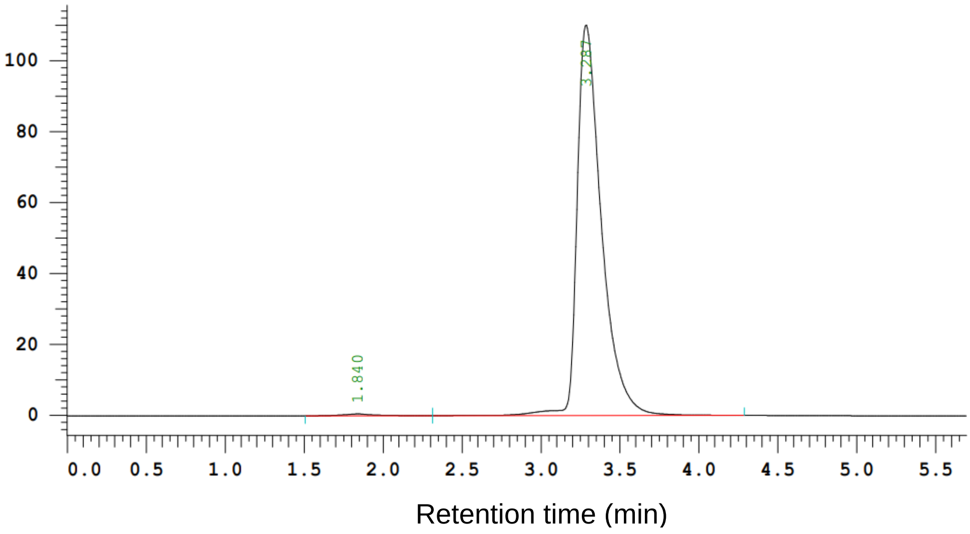

1.4 Spectra of compound **4c**.

^{1}H NMR spectrum

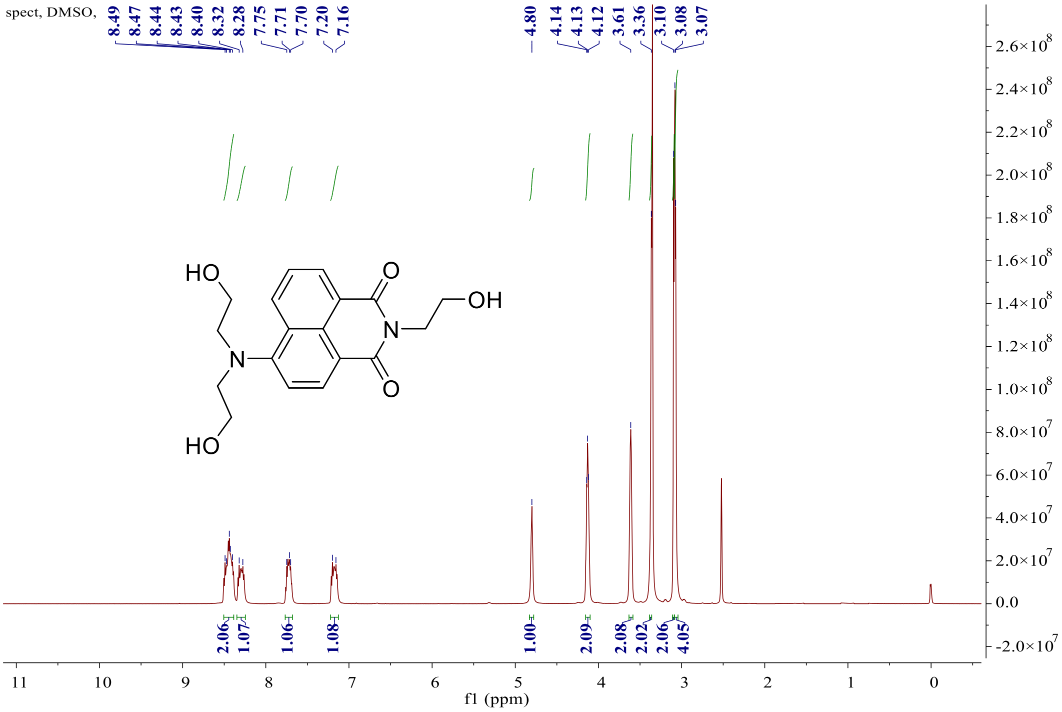

^{13}C NMR spectrum

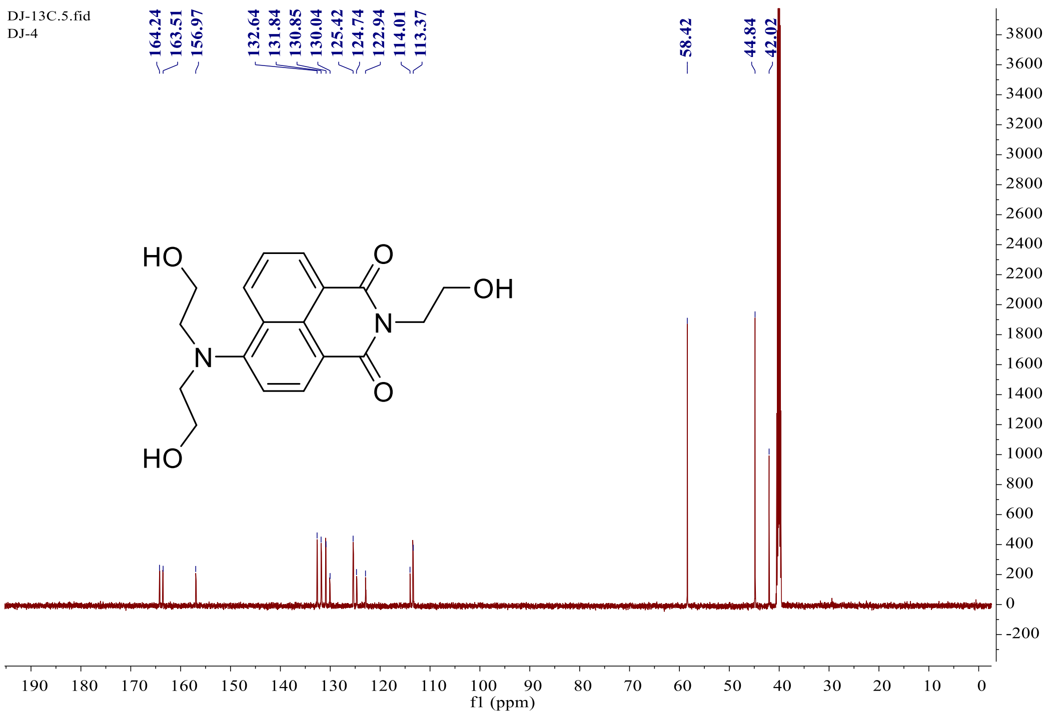

HPLC spectrum

No.	RT	Area (%)	Concentration (%)	BC
1	1. 807	4968	0. 270	BB
2	3. 293	1835807	99. 730	BB
		1840775	100. 000	

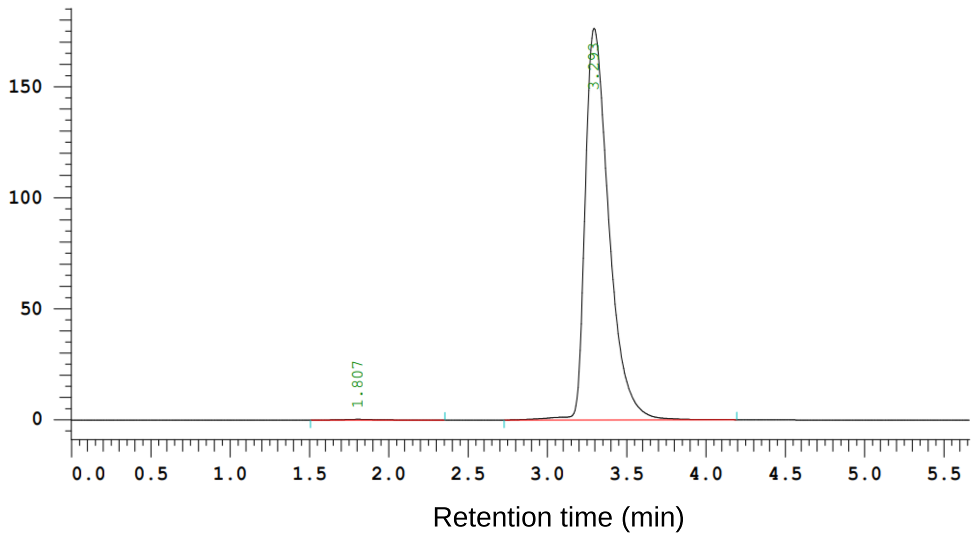

1.5 Spectra of compound **5**.

^{1}H NMR spectrum

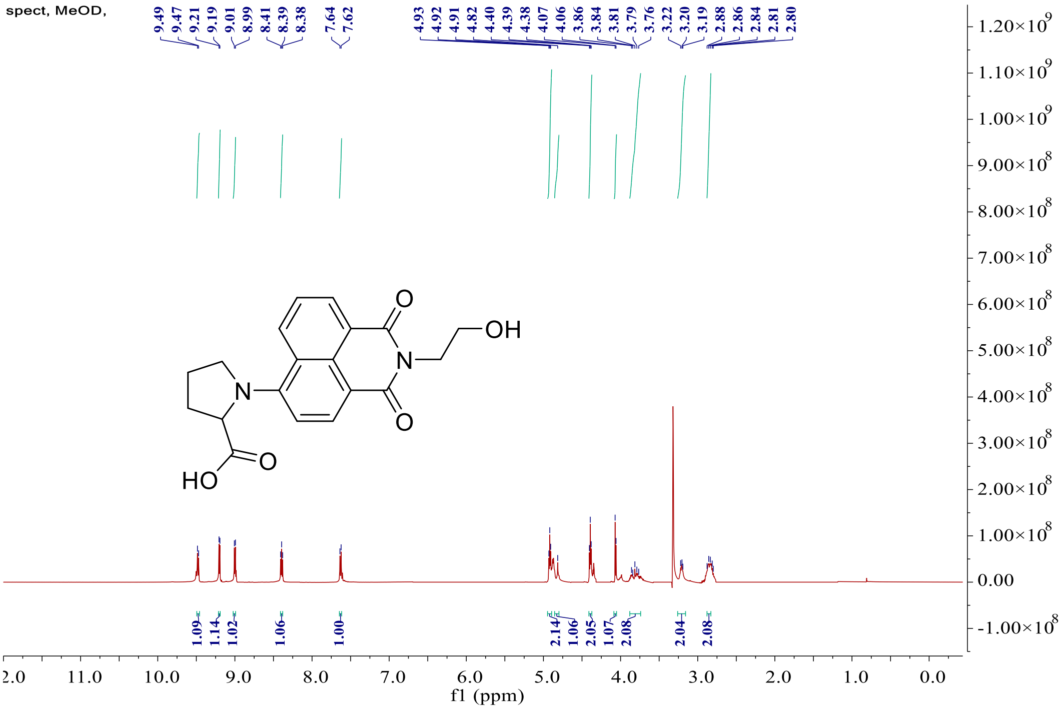

HRMS spectrum

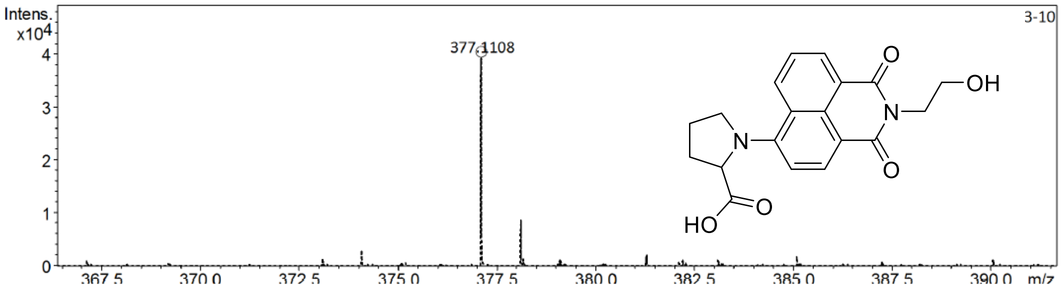

HPLC spectrum

No.	RT	Area (%)	Concentration (%)	BC
1	1. 927	17050	0. 444	BV
2	3. 293	3820240	99. 442	VV
3	4. 040	4378	0. 114	TBB
		3841668	100. 000	

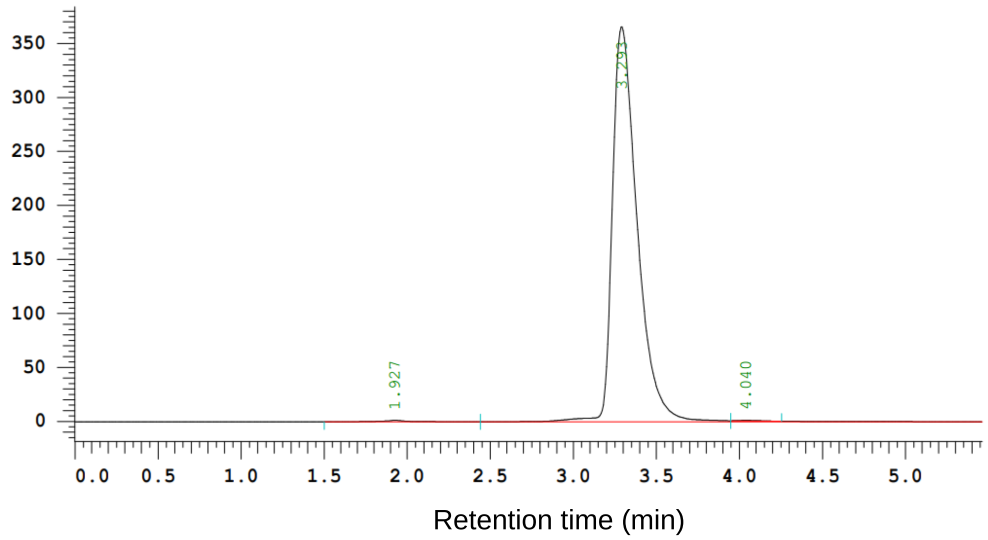

1.6 Spectra of compound **6a**.

^{1}H NMR spectrum

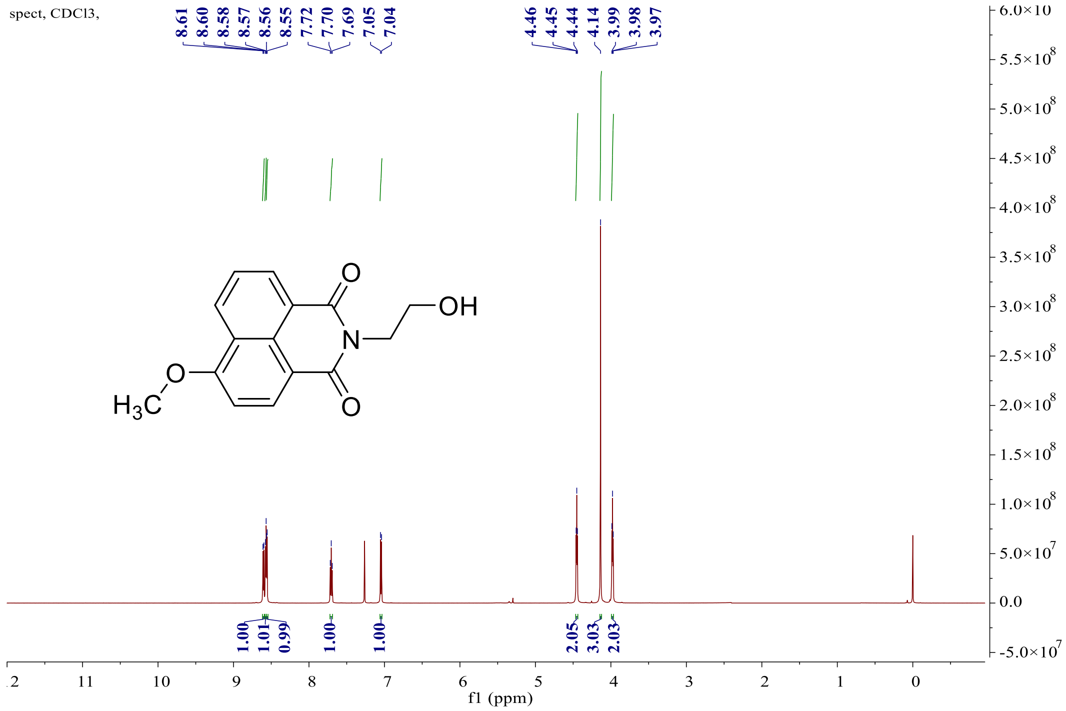

^{13}C NMR spectrum

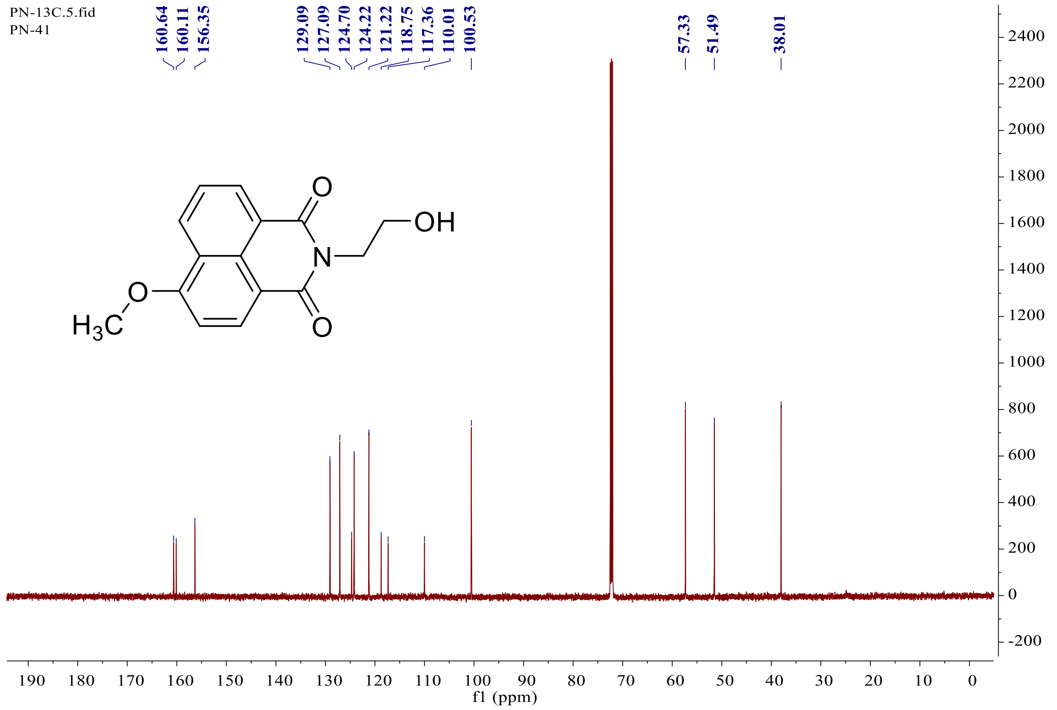

HRMS spectrum

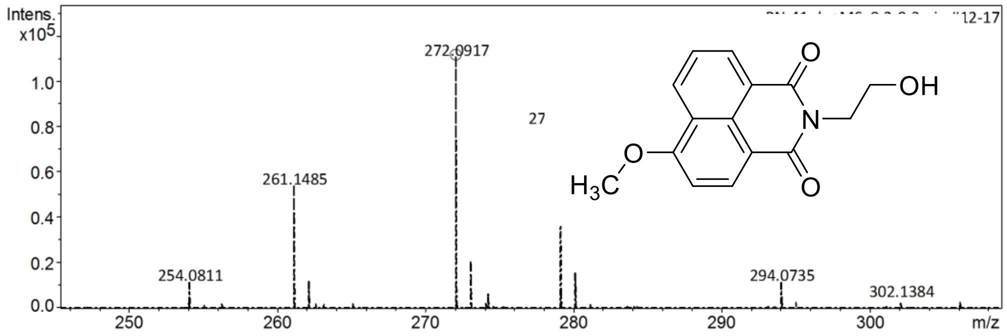

HPLC spectrum

No.	RT	Area (%)	Concentration (%)	BC
1	0. 740	1201	0. 009	BV
2	1. 040	2706	0. 019	VB
3	1. 760	3852	0. 027	BB
4	3. 493	13928568	99. 294	BV
5	4. 553	91253	0. 651	TBB
		14027580	100. 000	

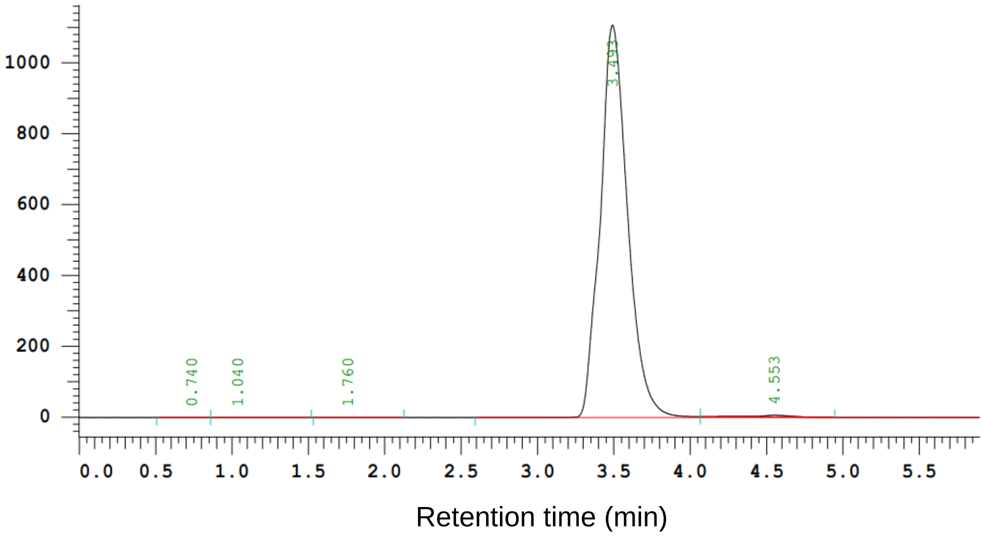

1.7 Spectra of compound **6c**.

^{1}H NMR spectrum

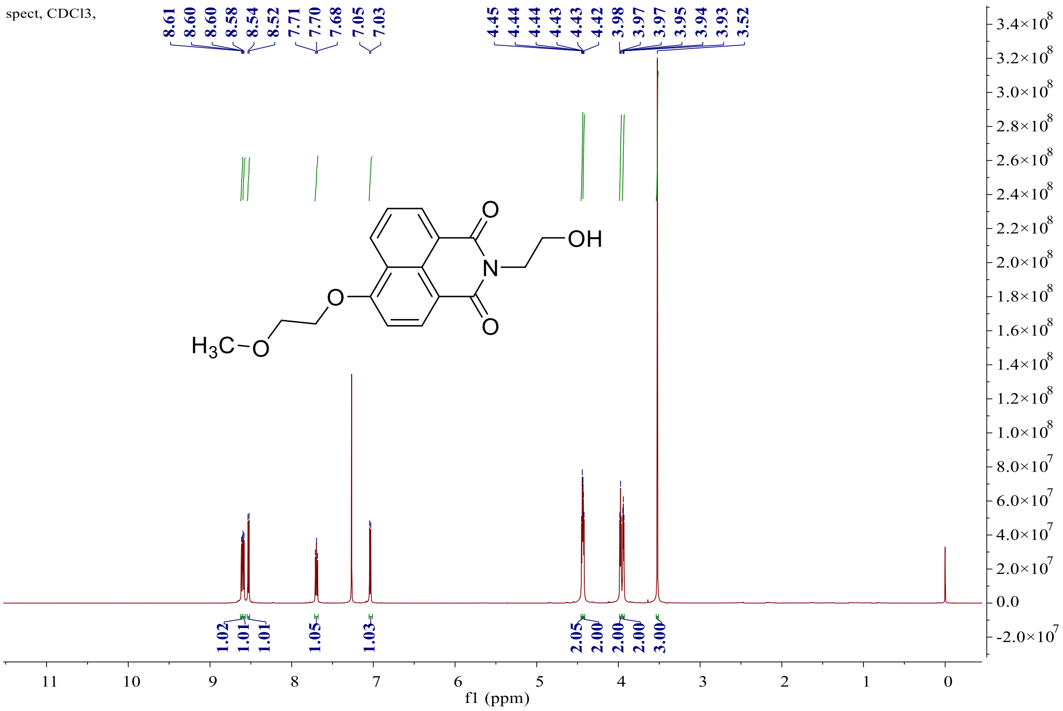

^{13}C NMR spectrum

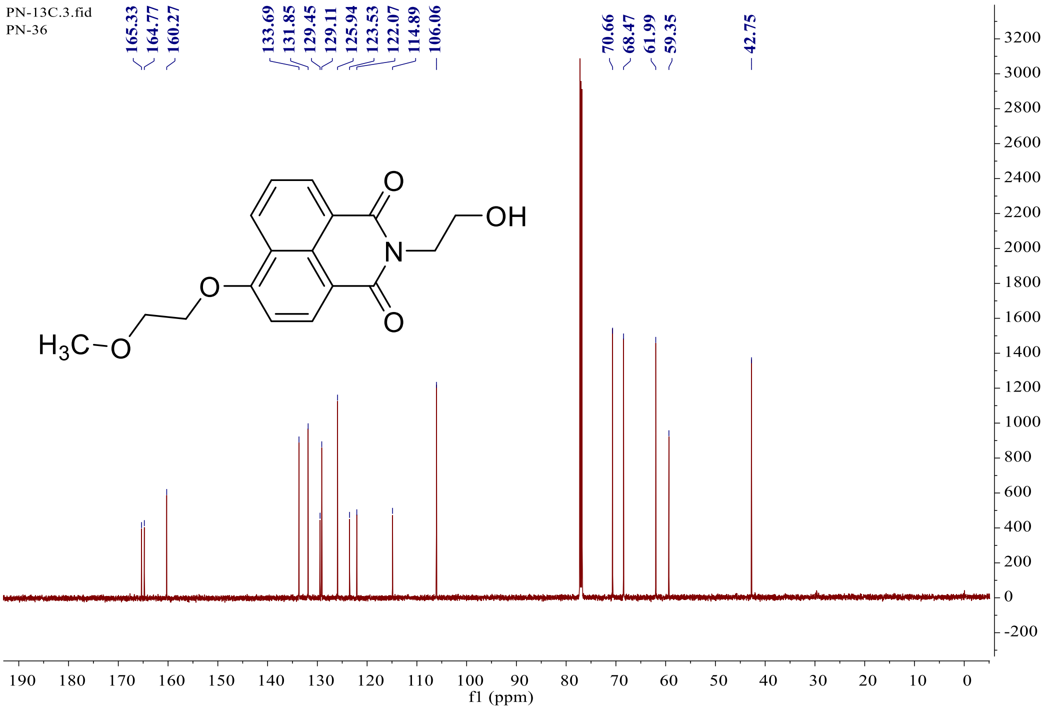

HRMS spectrum

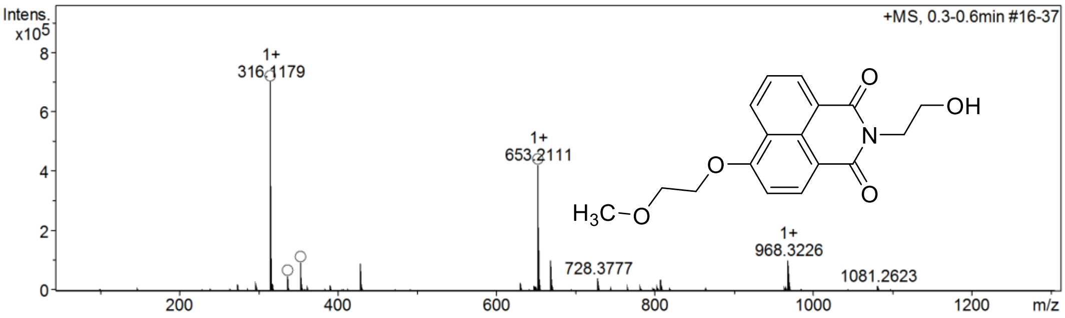

HPLC spectrum

No.	RT	Area (%)	Concentration (%)	BC
1	1. 780	1129	0. 110	BB
2	3. 467	1023672	99. 682	BB
3	4. 540	2132	0. 208	TBB
		1026933	100. 000	

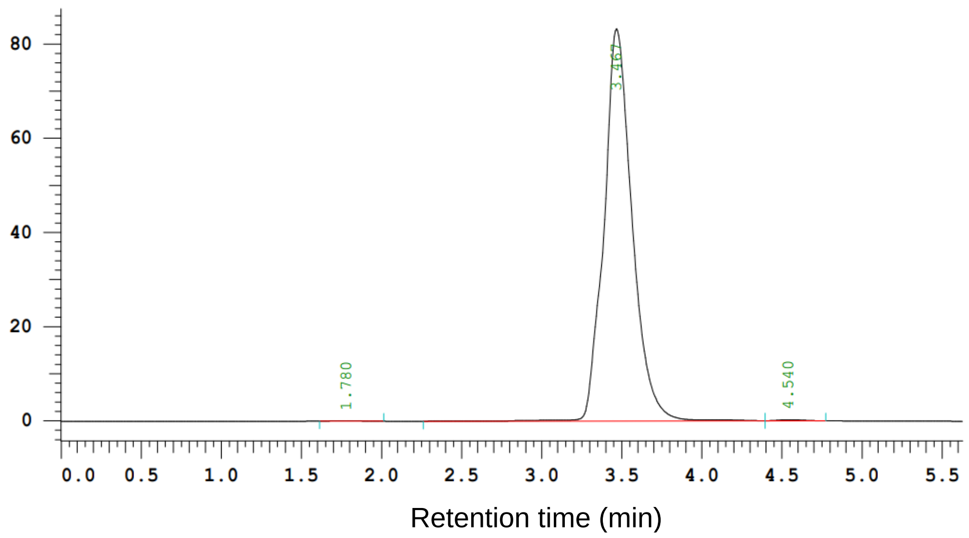

1.8 Spectra of compound **7a**.

^{1}H NMR spectrum

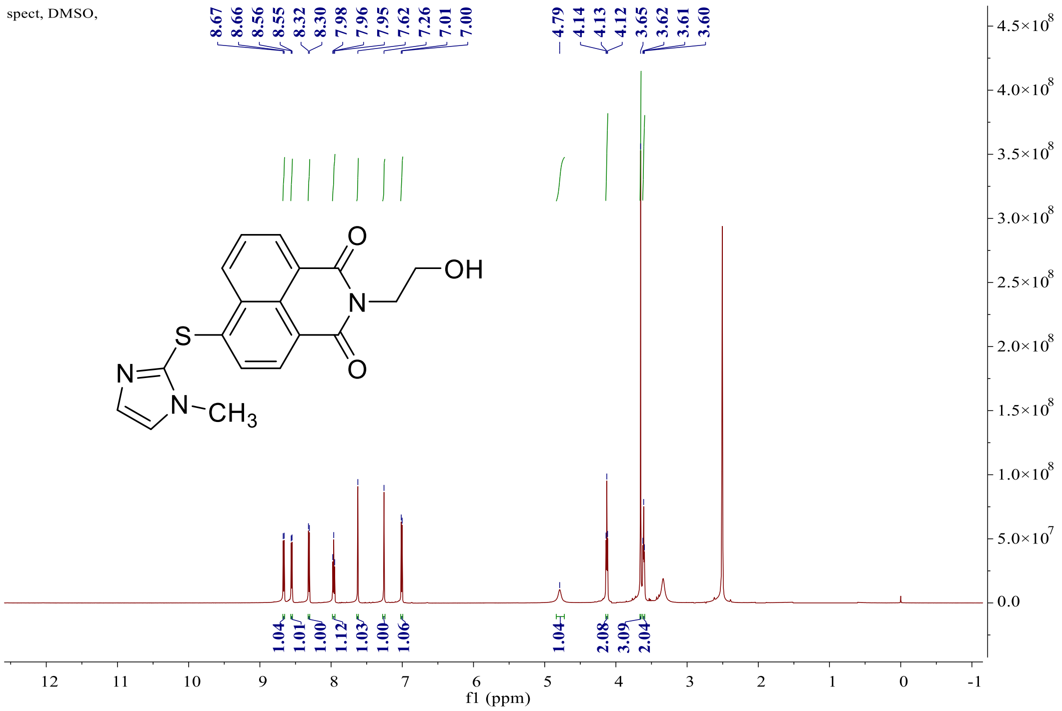

^{13}C NMR spectrum

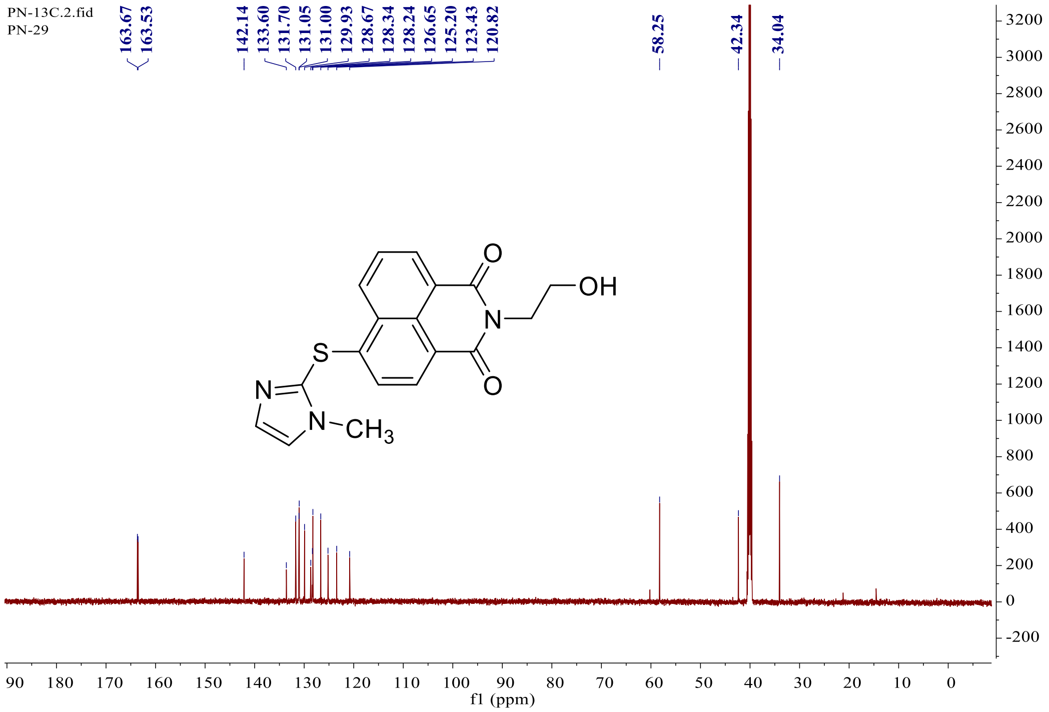

HRMS spectrum

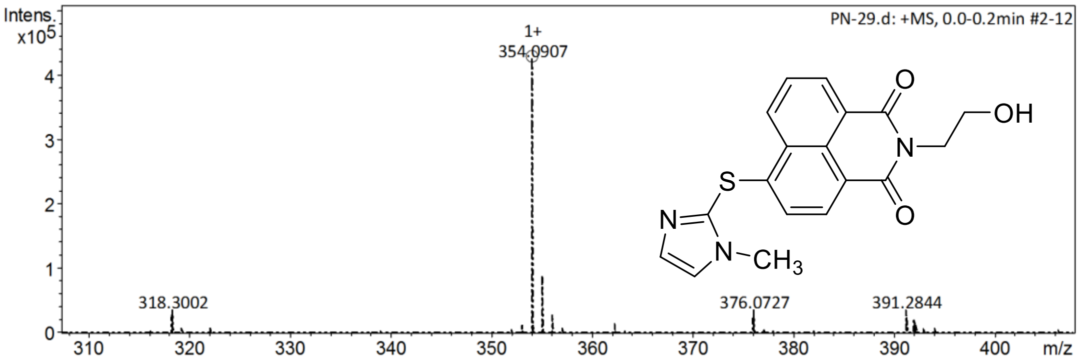

HPLC spectrum

No.	RT	Area (%)	Concentration (%)	BC
1	0. 740	1596	0. 008	BB
2	1. 820	5616	0. 028	BB
3	3. 440	19797858	99. 920	BV
4	4. 080	3352	0. 017	TBB
5	4. 873	5323	0. 027	TBB
		19813745	100. 000	

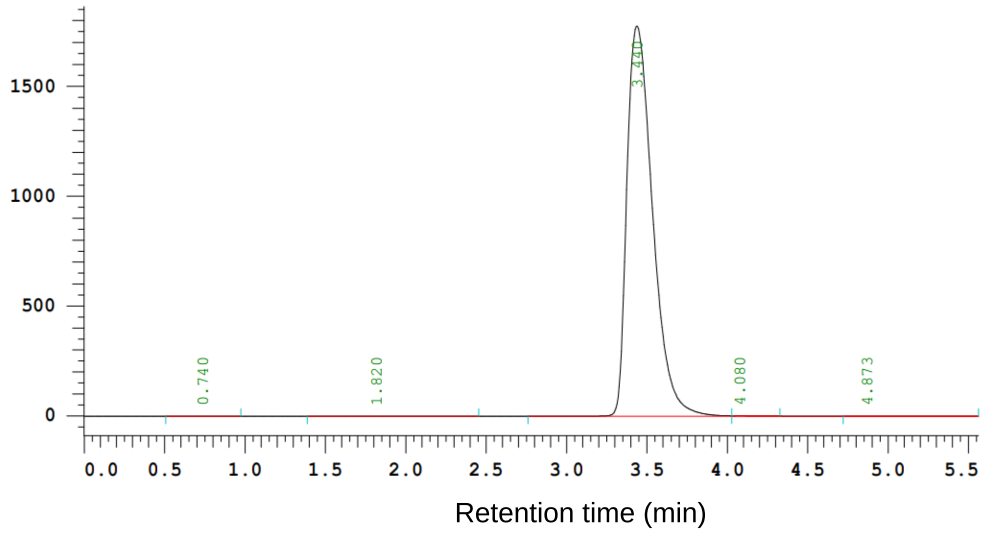

1.9 Spectra of compound **7b**.

^{1}H NMR spectrum

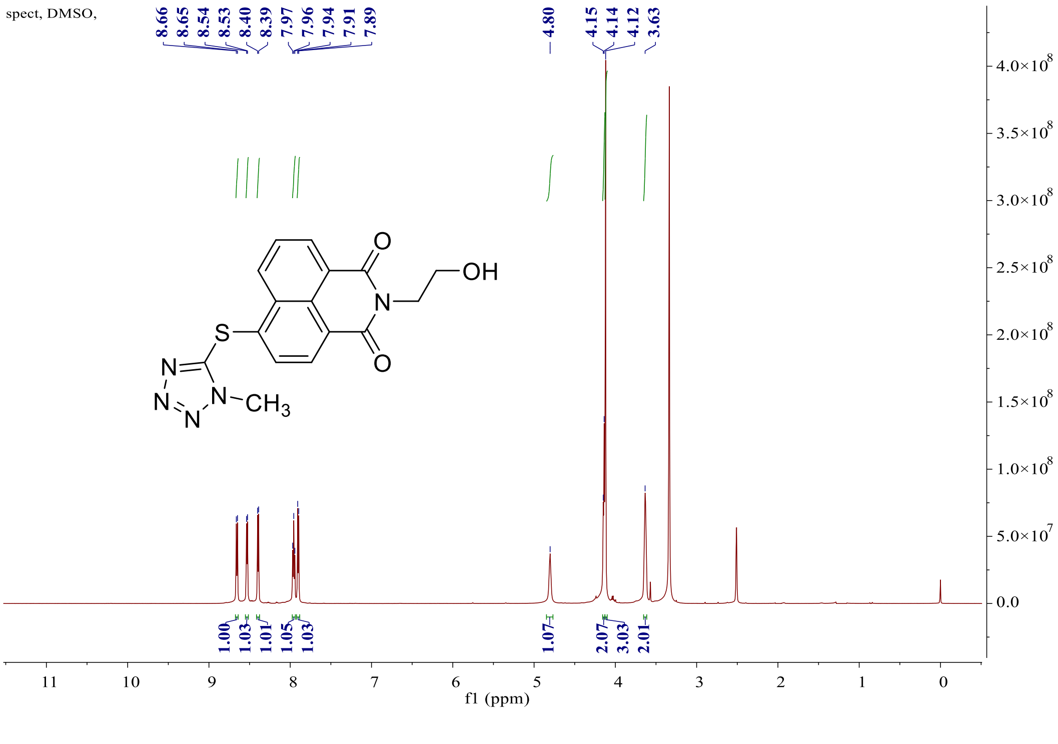

^{13}C NMR spectrum

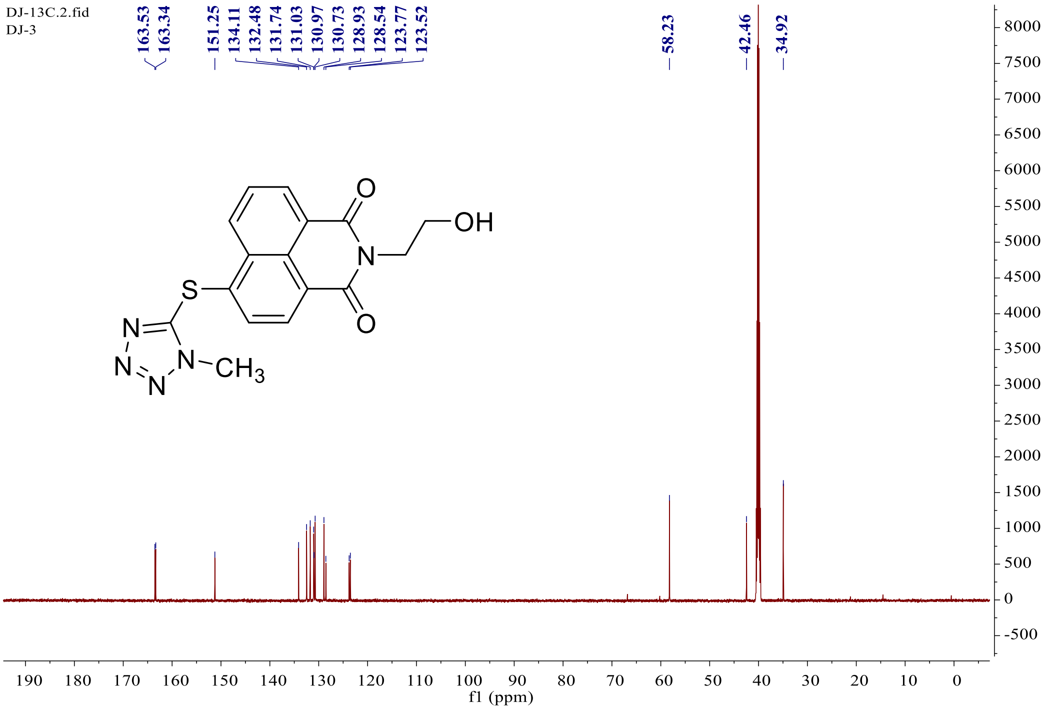

HRMS spectrum

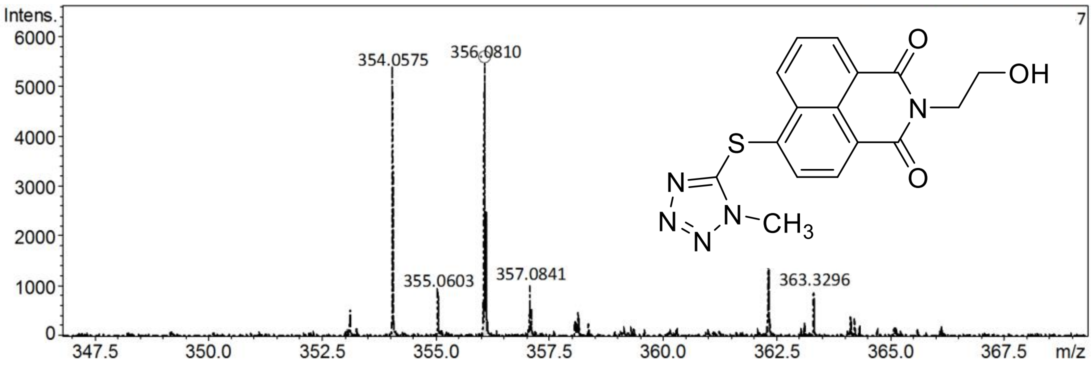

HPLC spectrum

No.	RT	Area (%)	Concentration (%)	BC
1	1. 820	5196	0. 027	BB
2	3. 433	19103287	99. 956	BV
3	4. 853	3124	0. 016	TBB
		19111607	100. 000	

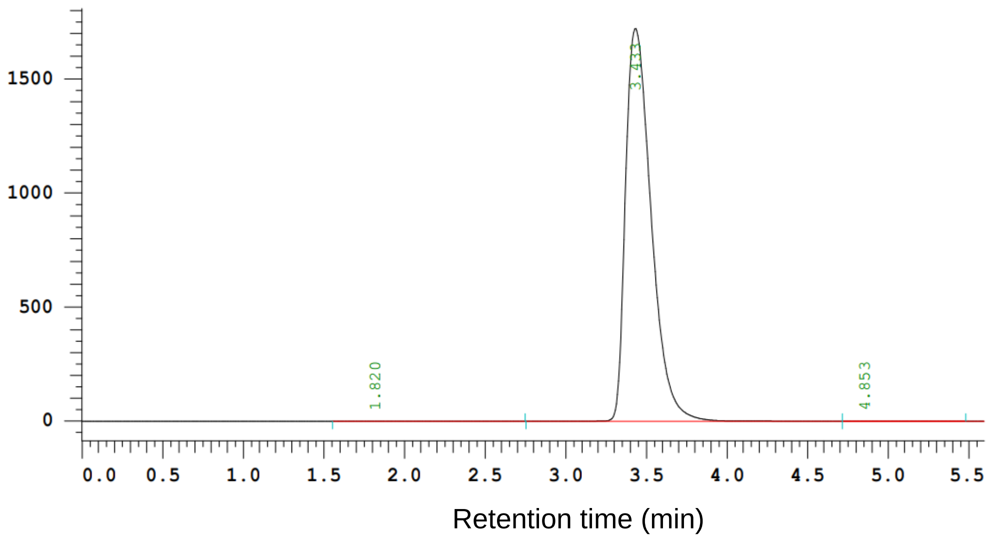

1.10 Spectra of compound **7c**.

^{1}H NMR spectrum

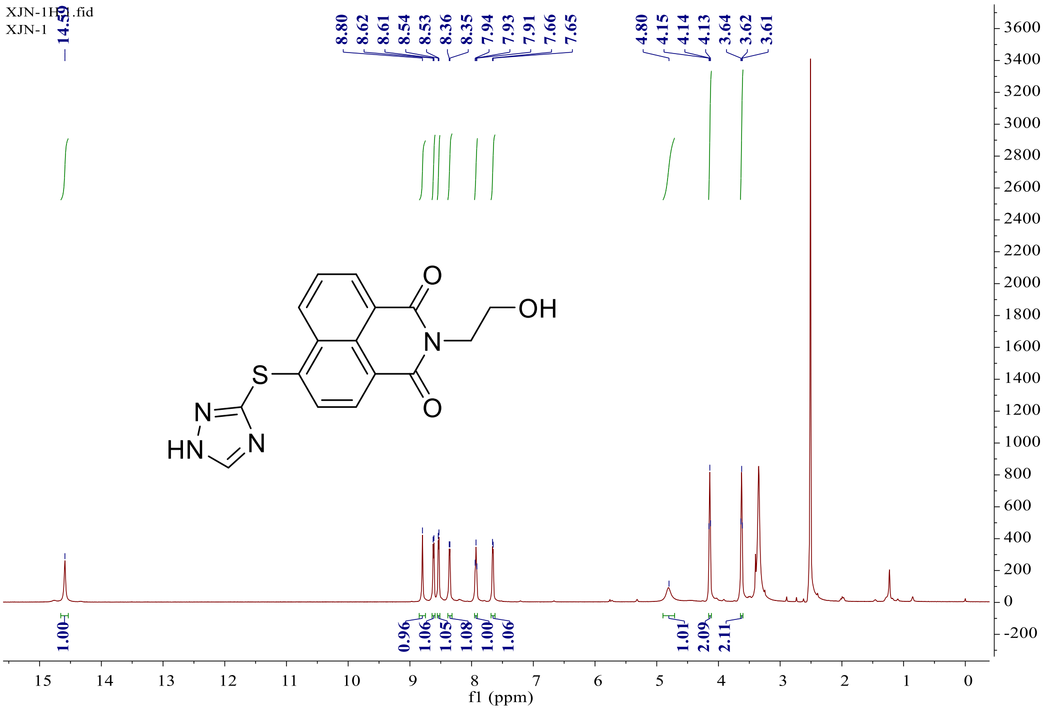

^{13}C NMR spectrum

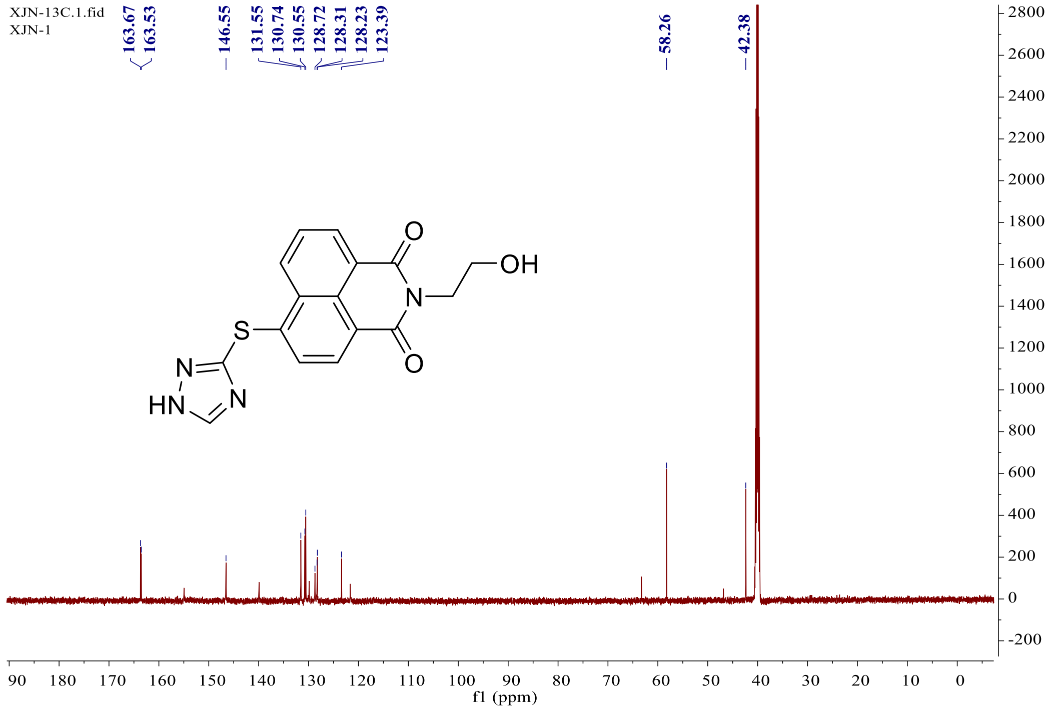

HRMS spectrum

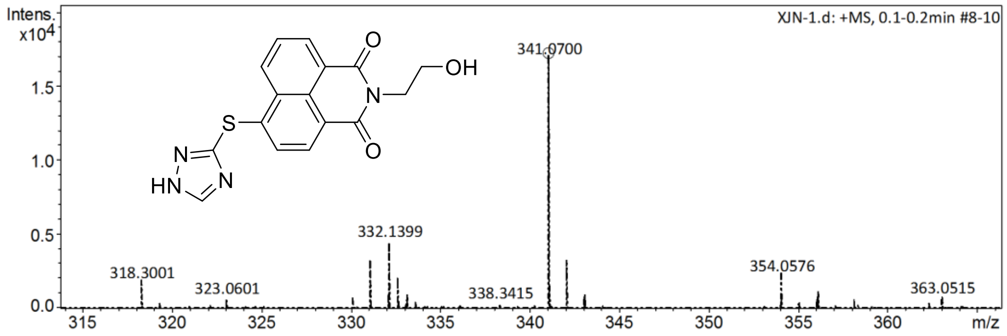

HPLC spectrum

No.	RT	Area (%)	Concentration (%)	BC
1	1. 840	3720	0. 022	BB
2	3. 427	16536450	99. 965	BV
3	4. 847	1995	0. 012	TBB
		16542165	100. 000	

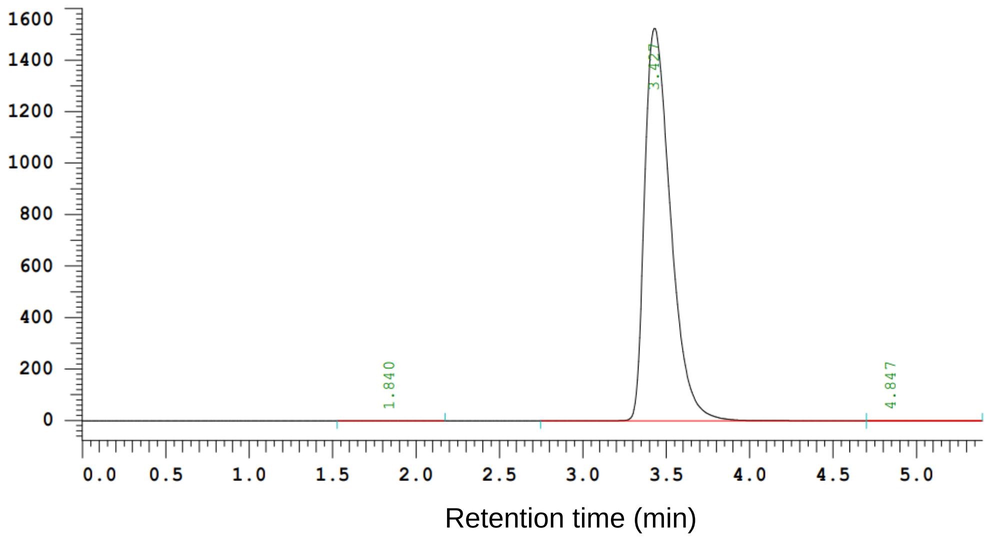

1.11 Spectra of compound **7d**.

^{1}H NMR spectrum

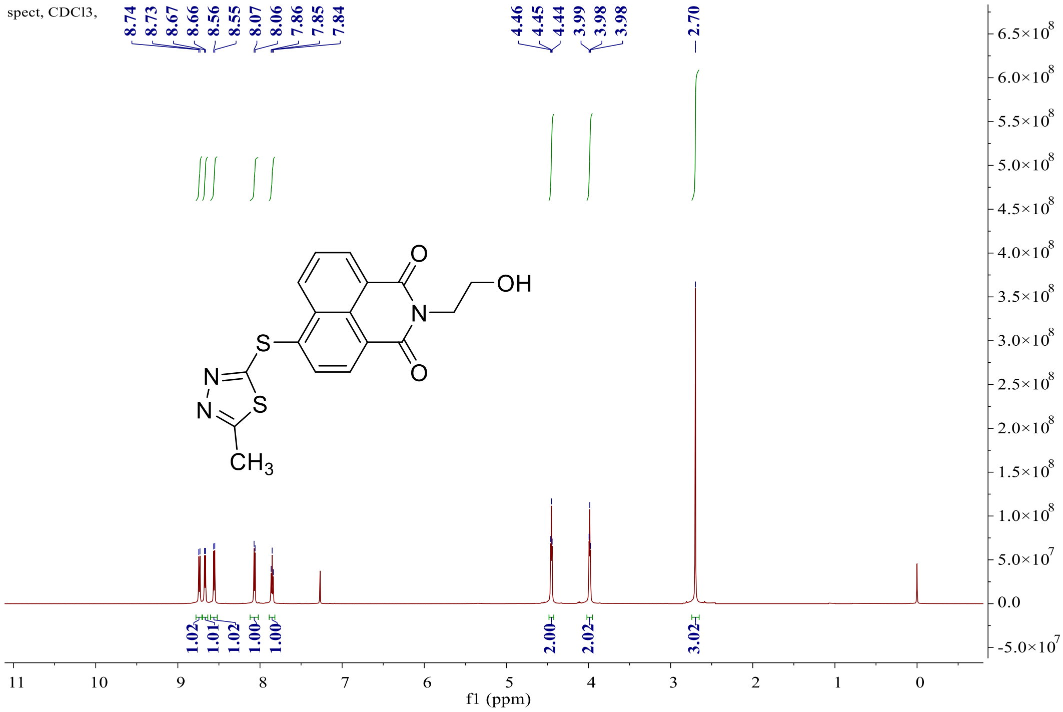

^{13}C NMR spectrum

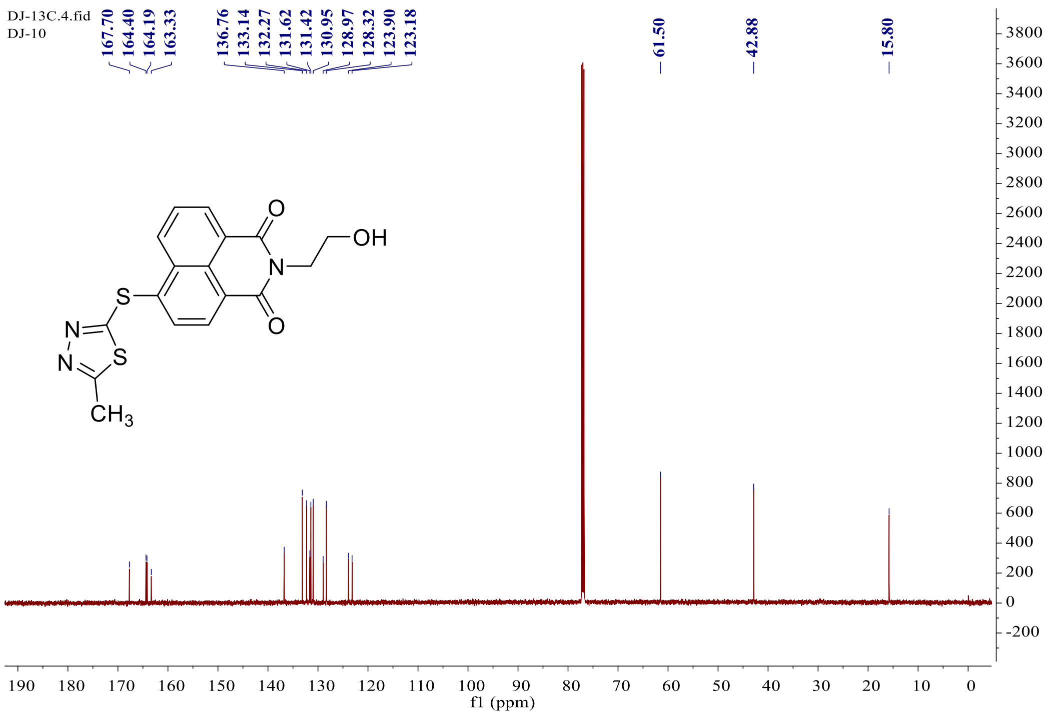

HRMS spectrum

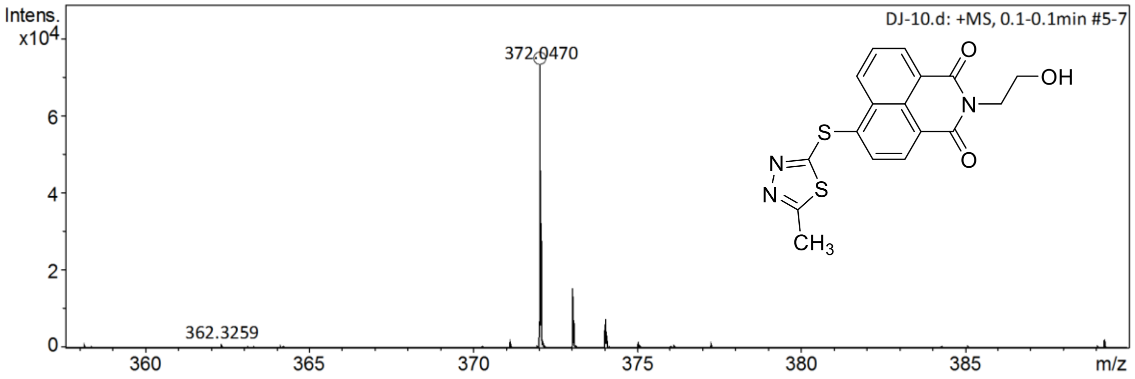

HPLC spectrum

No.	RT	Area (%)	Concentration (%)	BC
1	2. 173	4373	0. 038	BB
2	3. 427	11567703	99. 949	BV
3	4. 860	1490	0. 013	TBB
		11573566	100. 000	

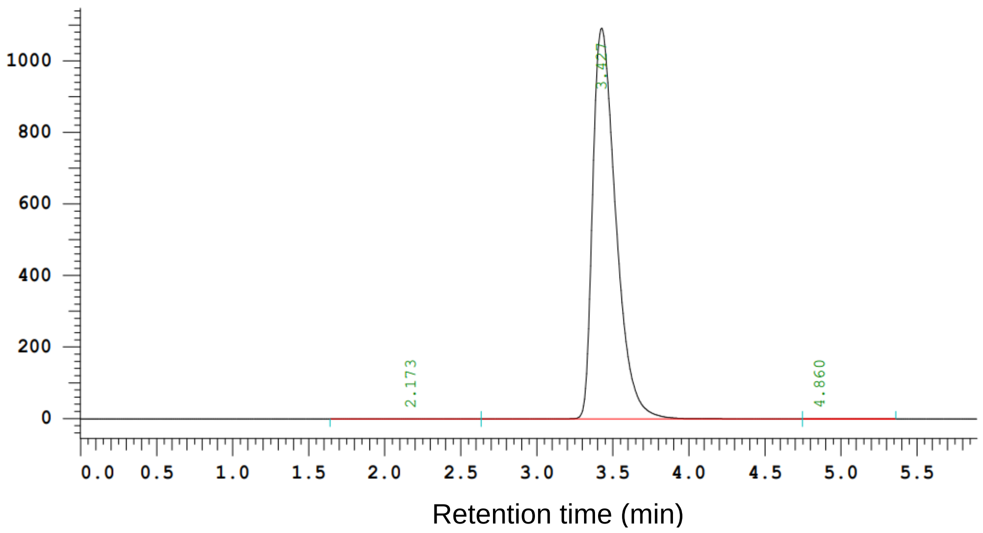

1.12 Spectra of compound **7e**.

^{1}H NMR spectrum

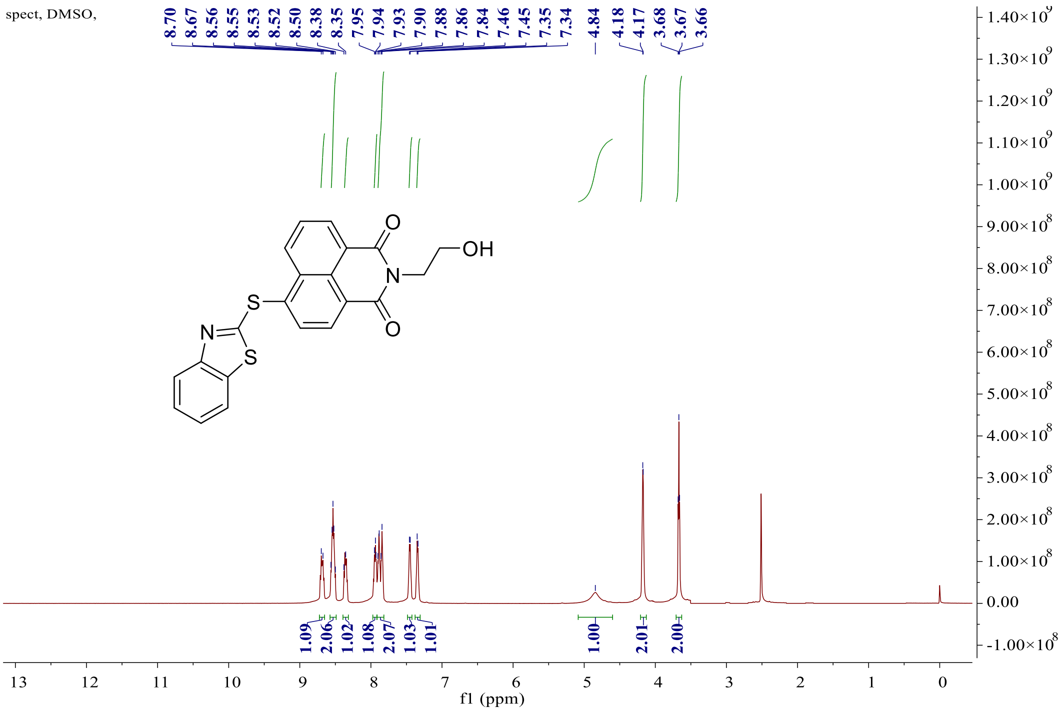

^{13}C NMR spectrum

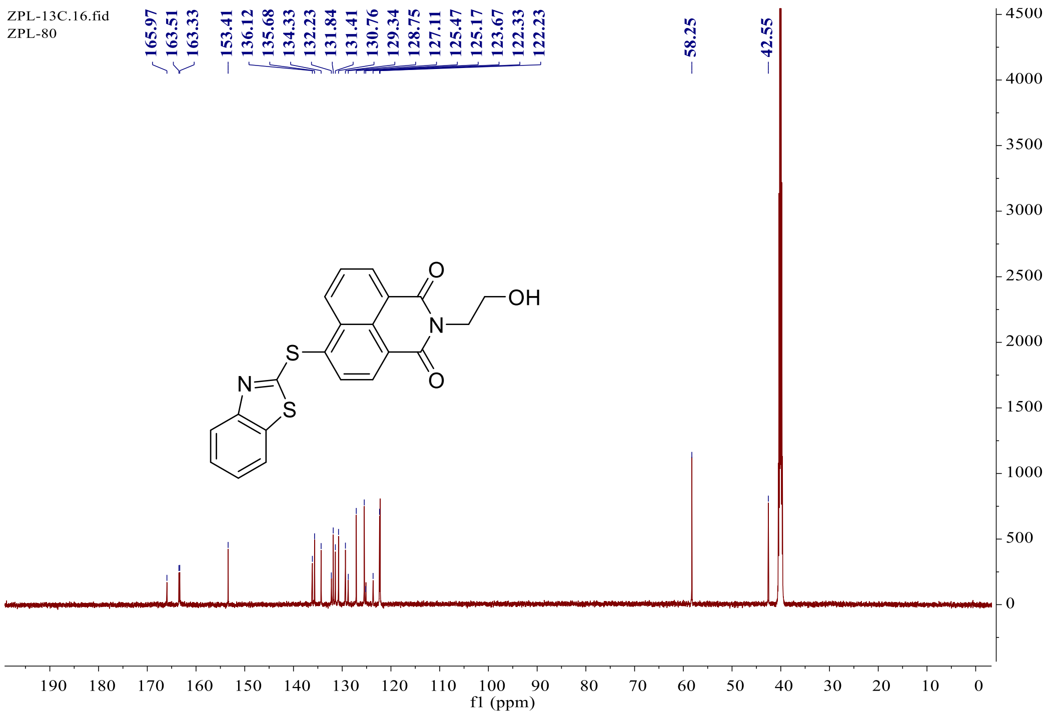

HRMS spectrum

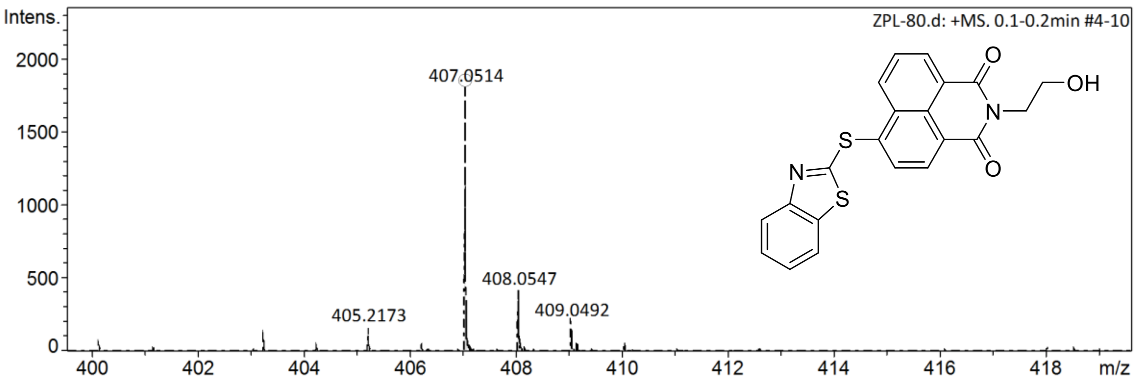

HPLC spectrum

No.	RT	Area (%)	Concentration (%)	BC
1	1. 787	7092	0. 335	BB
2	3. 133	17028	0. 805	BV
3	3. 427	2089339	98. 757	VV
4	4. 093	1924	0. 091	TBB
5	6. 433	247	0. 012	BB
		2115630	100. 000	

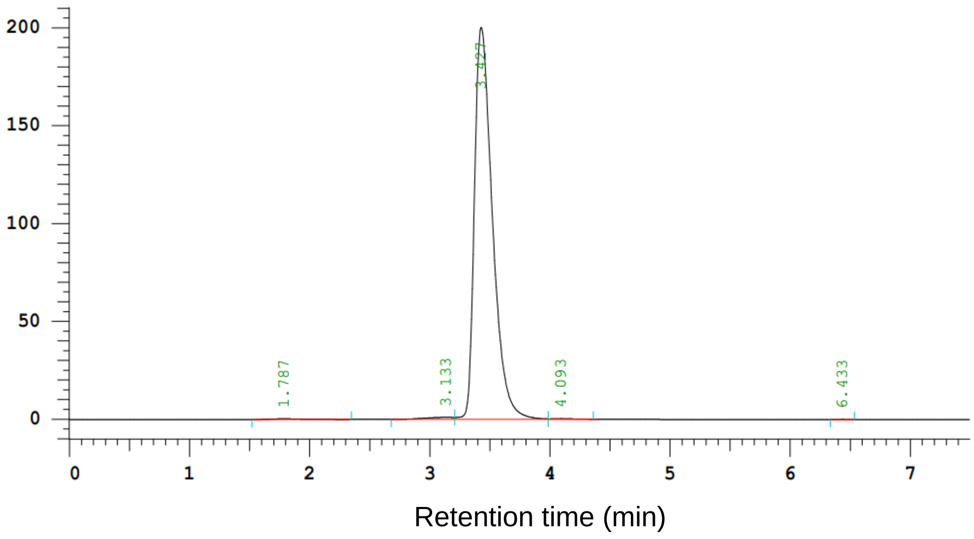

1.13 Spectra of compound **7f**.

^{1}H NMR spectrum

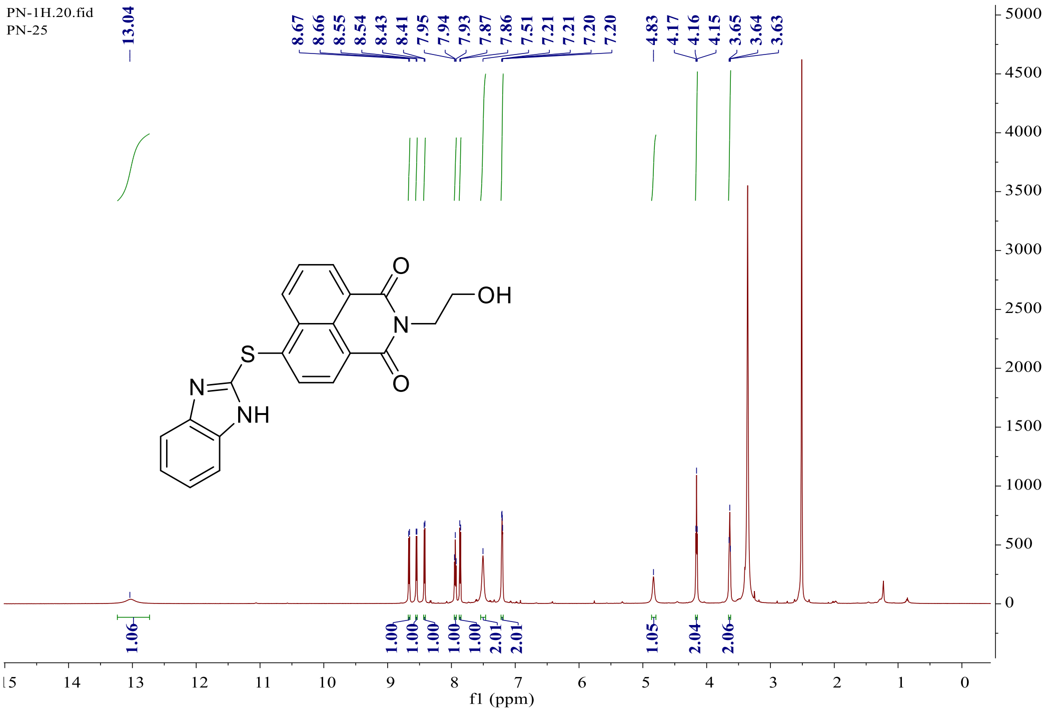

^{13}C NMR spectrum

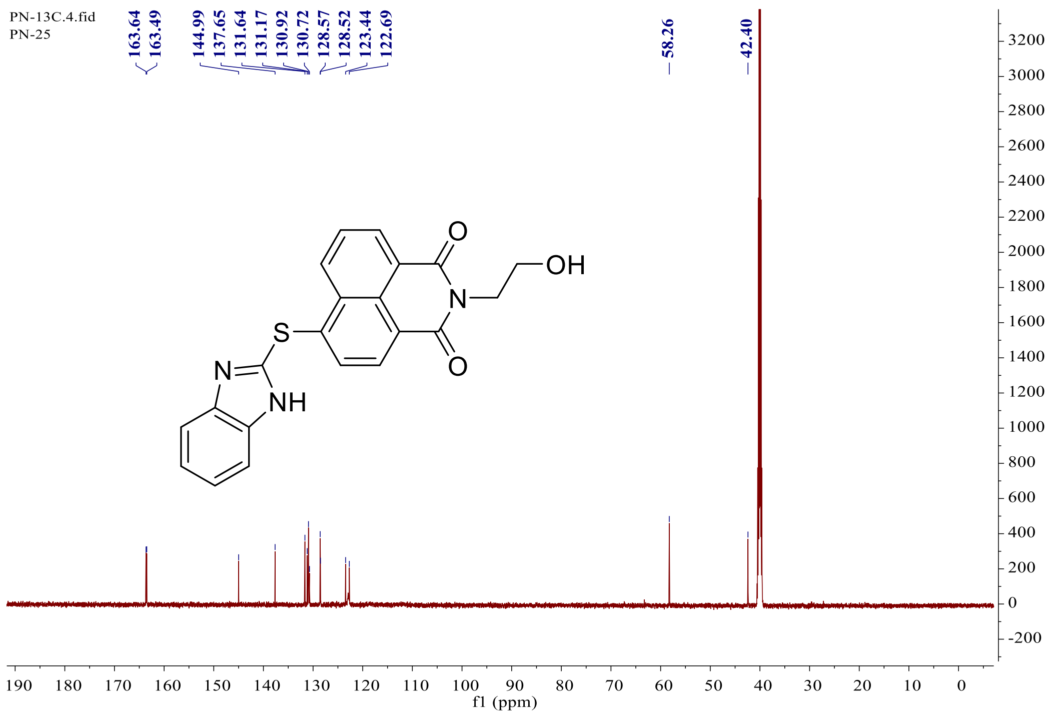

HRMS spectrum

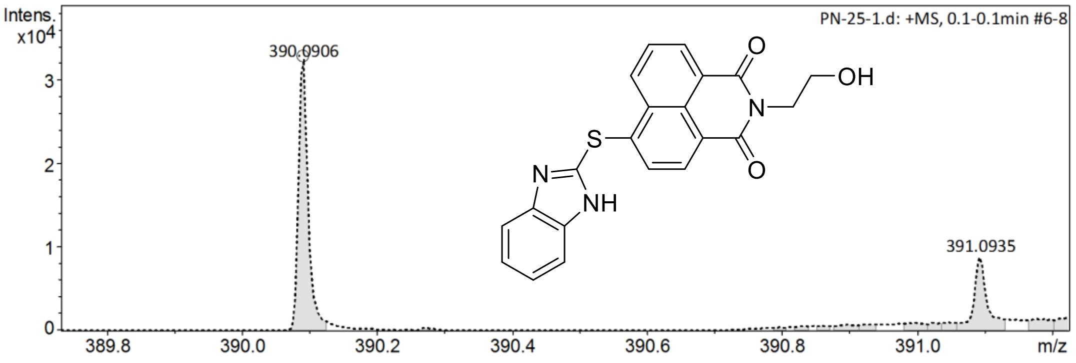

HPLC spectrum

No.	RT	Area (%)	Concentration (%)	BC
1	1. 200	1445	0. 016	BB
2	3. 127	8031	0. 088	BV
3	3. 420	9163452	99. 897	VB
		9172928	100. 000	

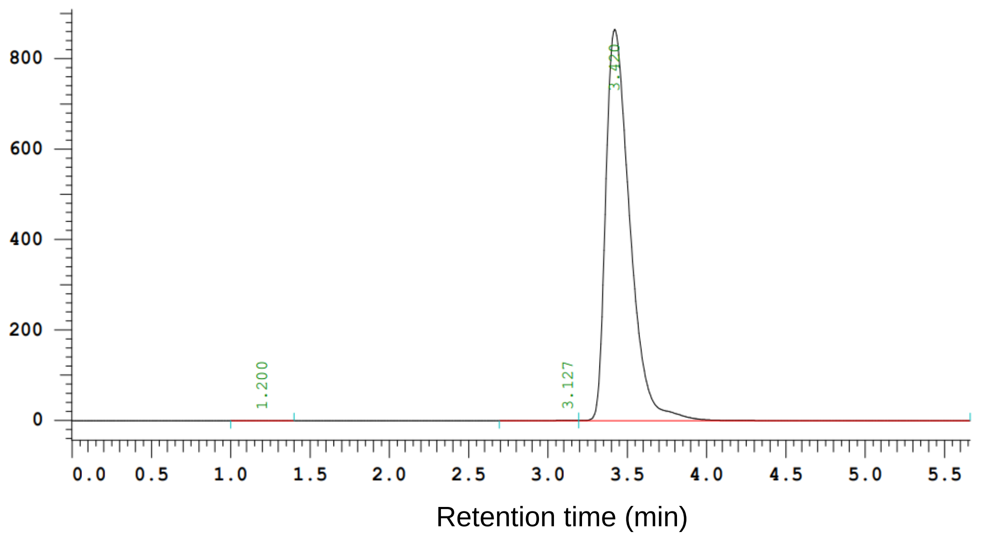

1.14 Spectra of compound **8a**.

^{1}H NMR spectrum

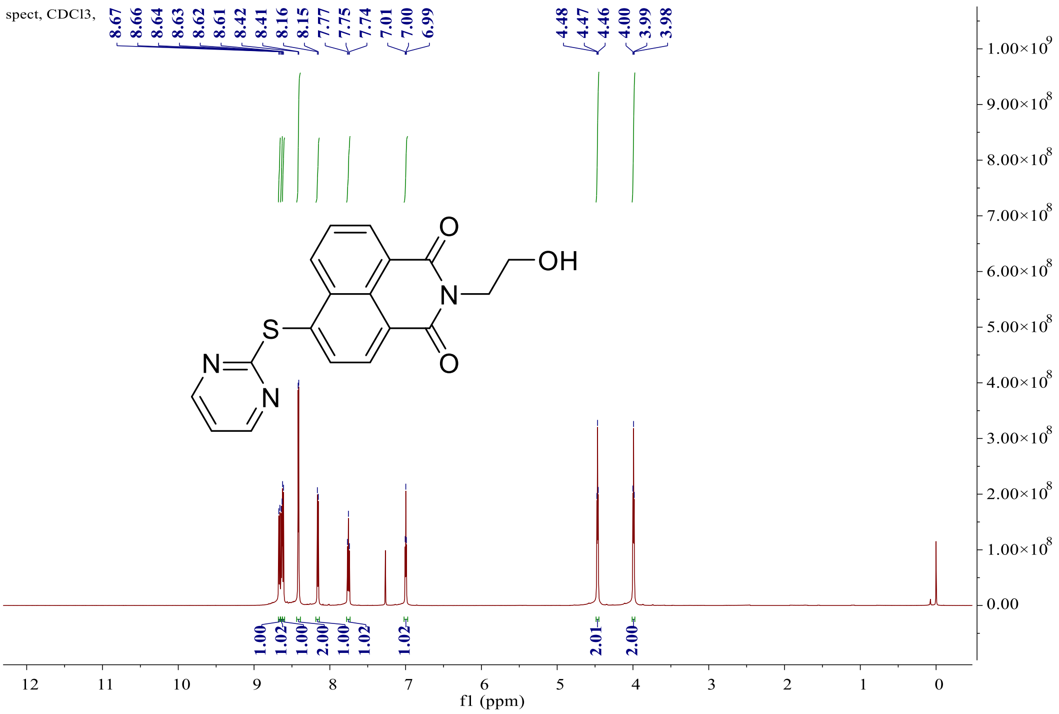

^{13}C NMR spectrum

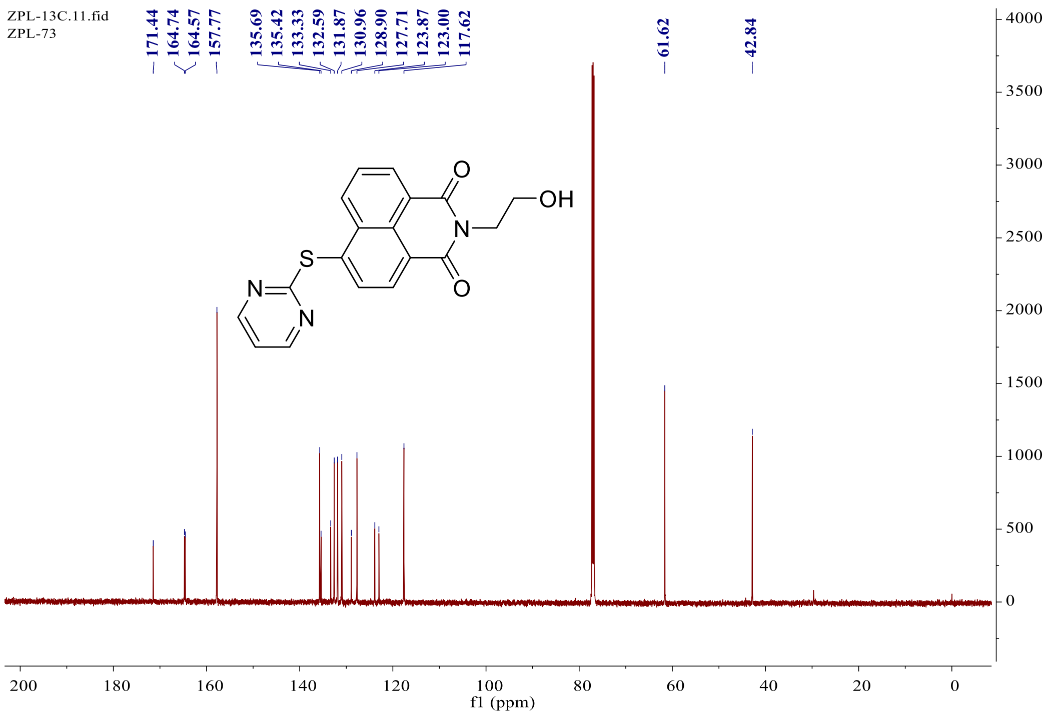

HRMS spectrum

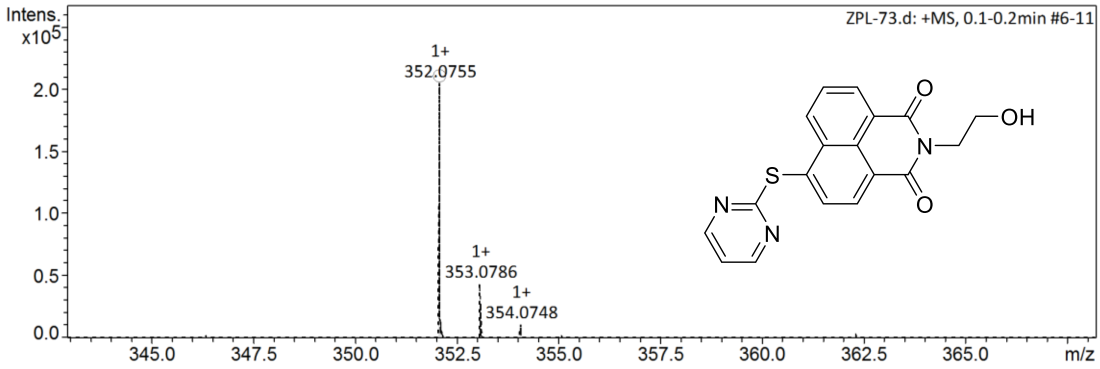

HPLC spectrum

No.	RT	Area (%)	Concentration (%)	BC
1	1. 833	1817	0. 011	BB
2	2. 460	484	0. 003	BB
3	3. 133	18422	0. 108	BV
4	3. 427	17013696	99. 878	VB
		17034419	100. 000	

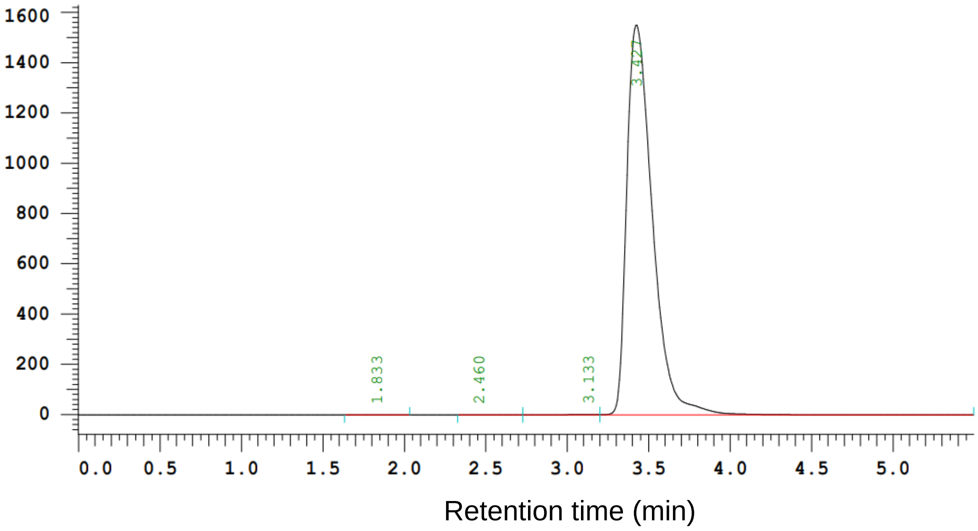

1.15 Spectra of compound **8b**.

^{1}H NMR spectrum

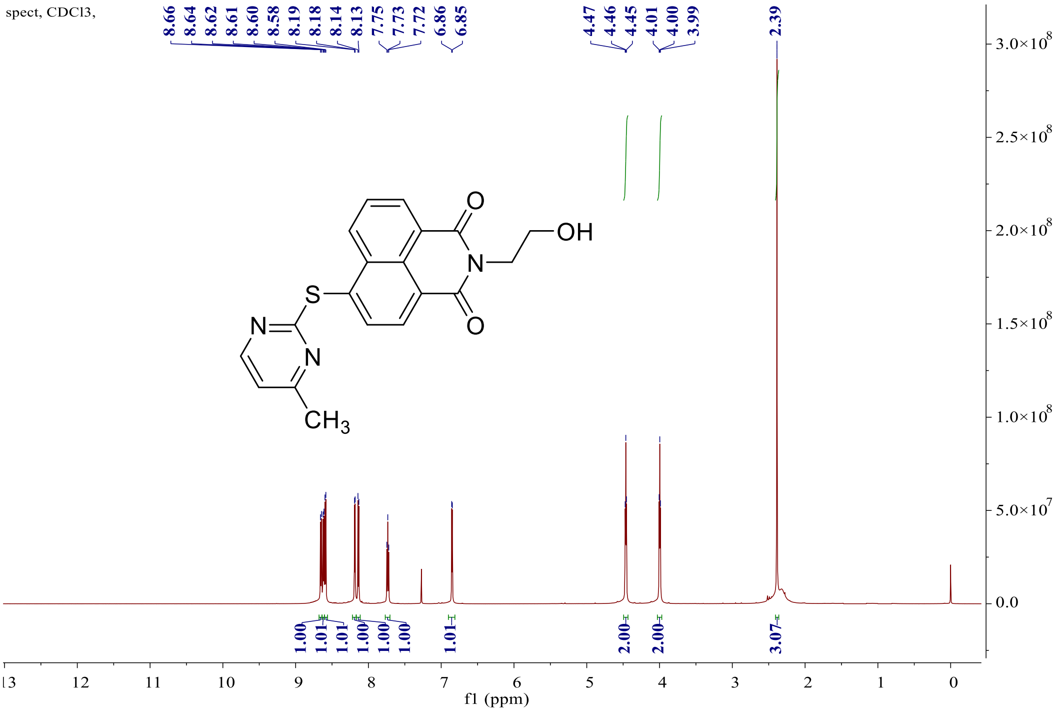

^{13}C NMR spectrum

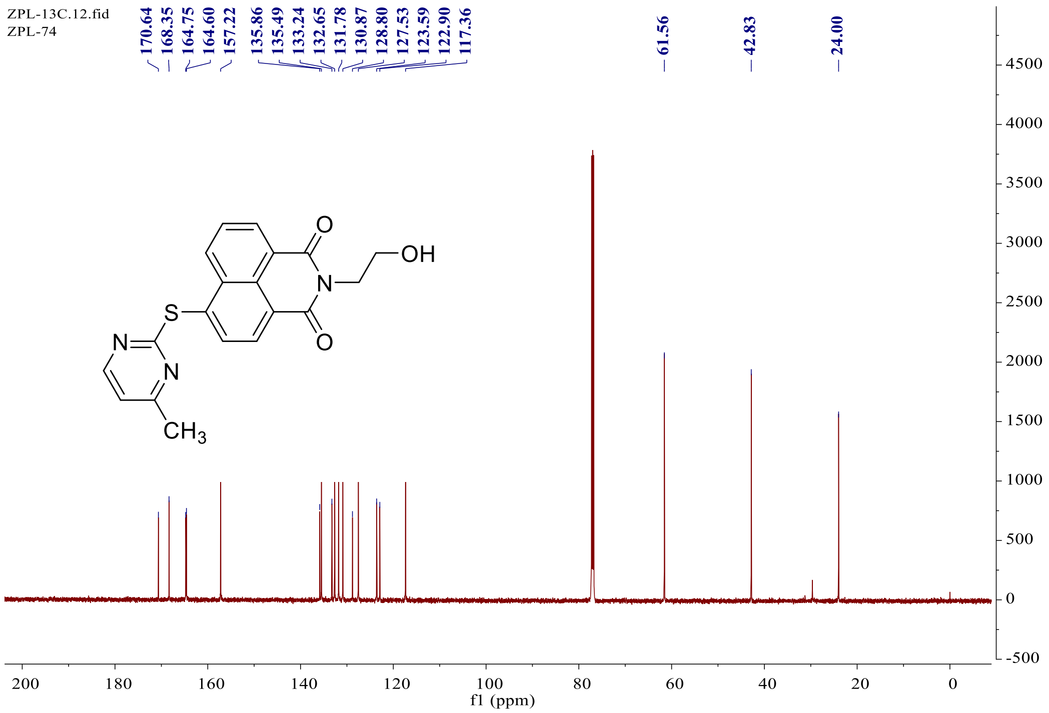

HRMS spectrum

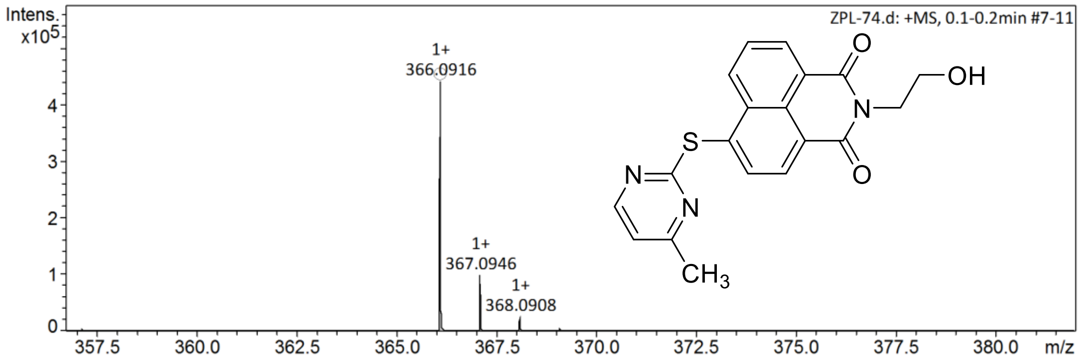

HPLC spectrum

No.	RT	Area (%)	Concentration (%)	BC
1	1. 893	6700	0. 073	BB
2	3. 187	9098760	99. 461	BV
3	3. 747	42581	0. 465	TBB
		9148041	100. 000	

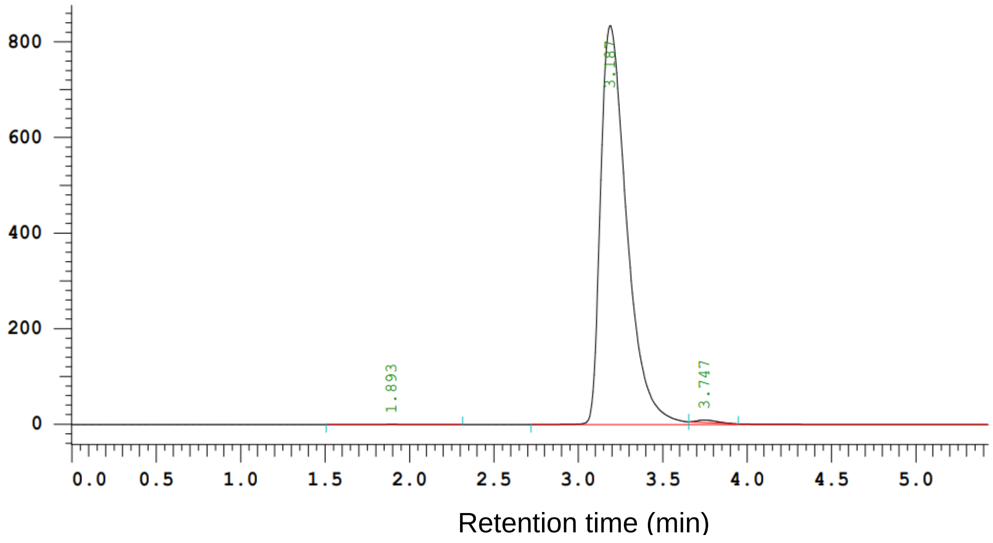

1.16 Spectra of compound **8c**.

^{1}H NMR spectrum

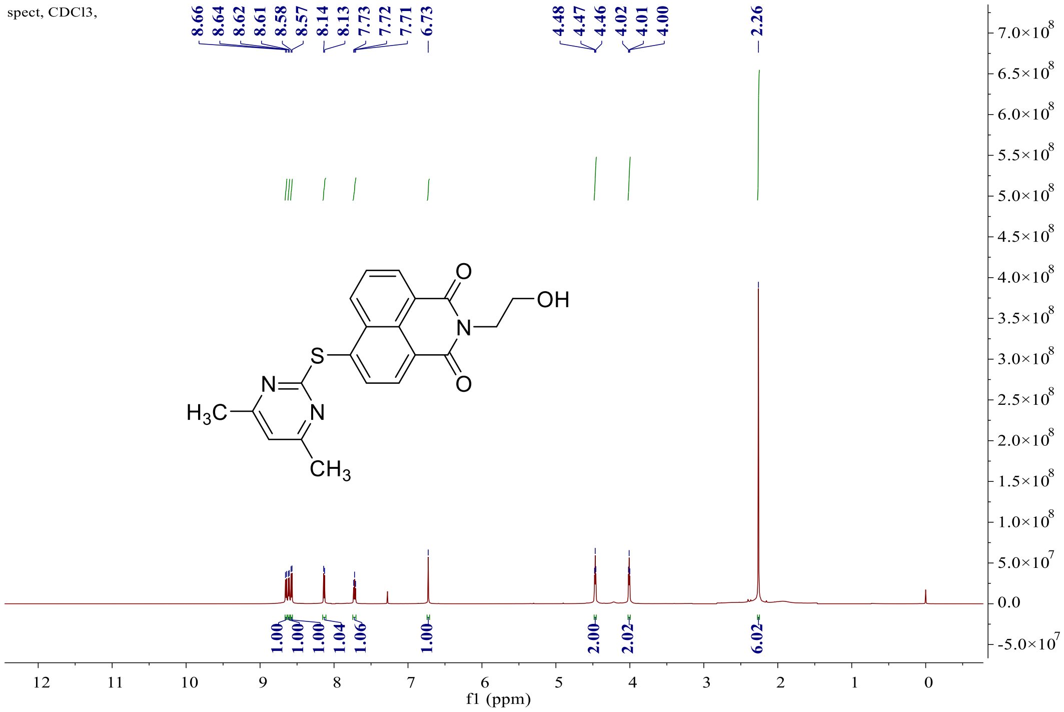

^{13}C NMR spectrum

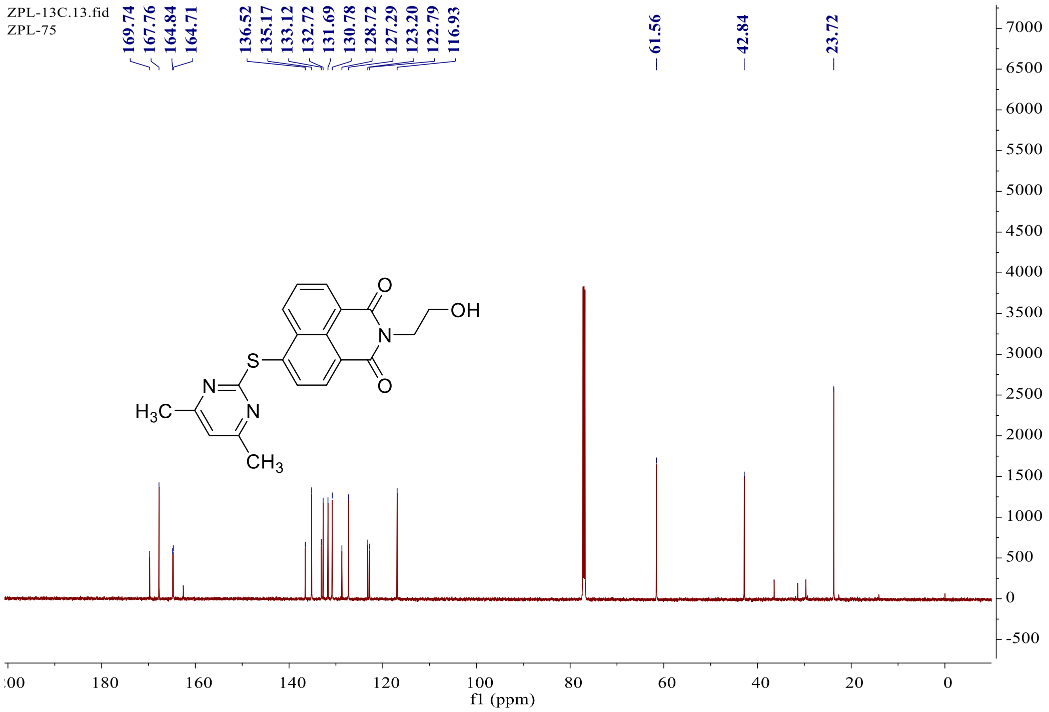

HRMS spectrum

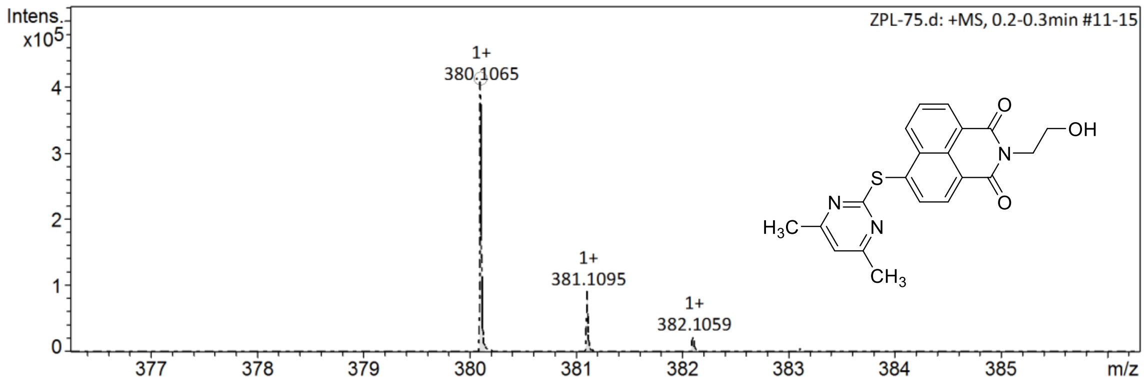

HPLC spectrum

No.	RT	Area (%)	Concentration (%)	BC
1	1. 867	2945	0. 040	BB
2	2. 540	948	0. 013	BB
3	3. 193	7276545	99. 472	BV
4	3. 747	34736	0. 475	TBB
		7315174	100. 000	

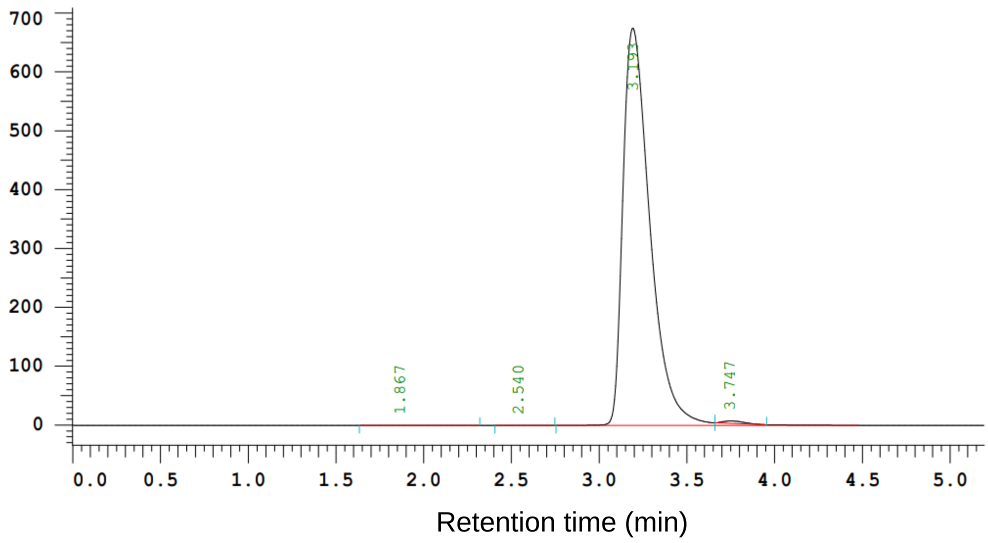

1.17 Spectra of compound **8d**.

^{1}H NMR spectrum

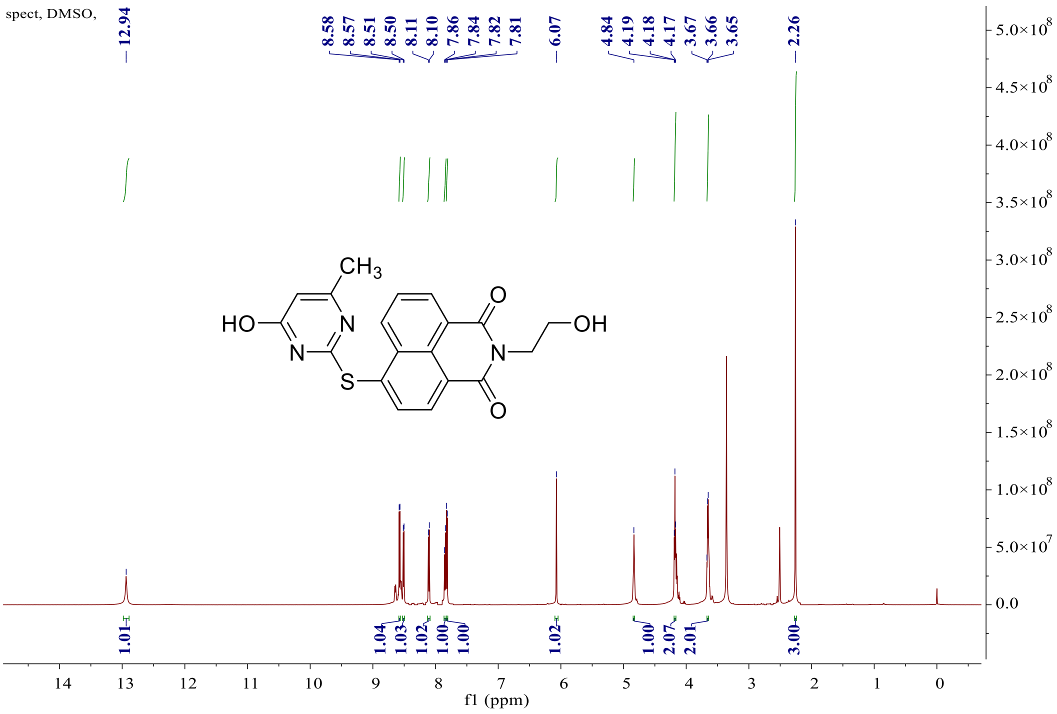

^{13}C NMR spectrum

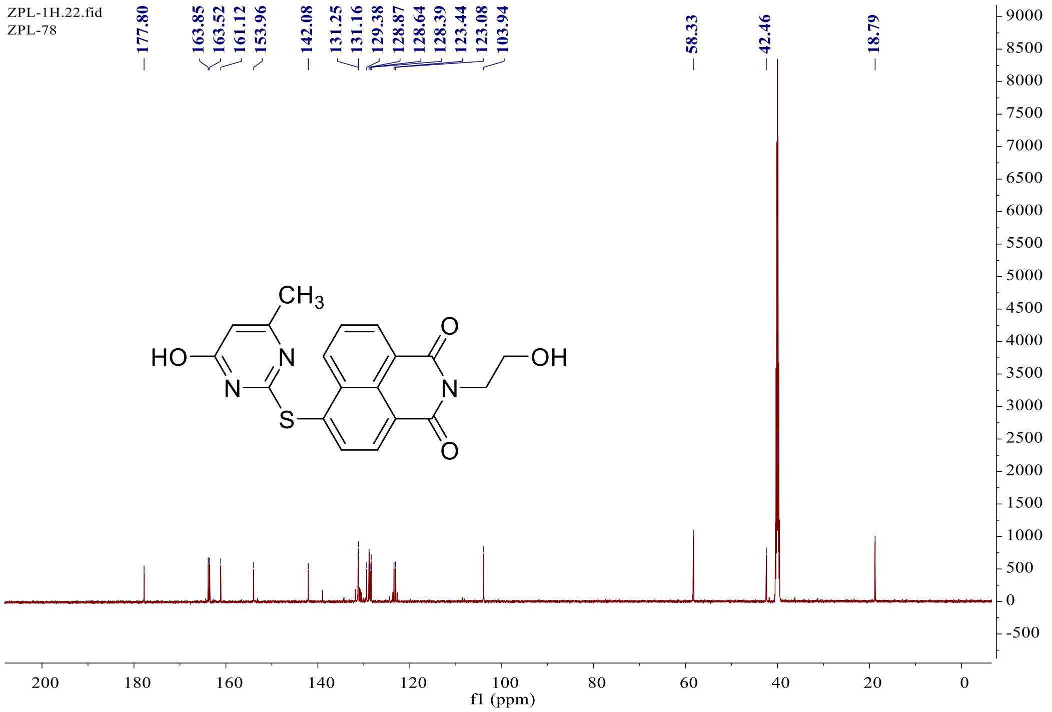

HRMS spectrum

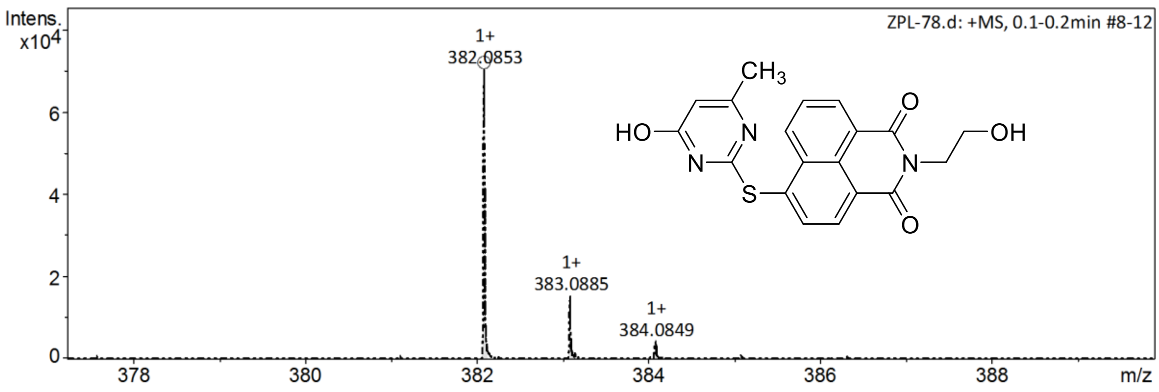

HPLC spectrum

No.	RT	Area (%)	Concentration (%)	BC
1	0. 233	1983	0. 025	BB
2	1. 860	1732	0. 022	BB
3	3. 193	7995820	99. 473	BV
4	3. 753	38670	0. 481	TBB
		8038205	100. 000	

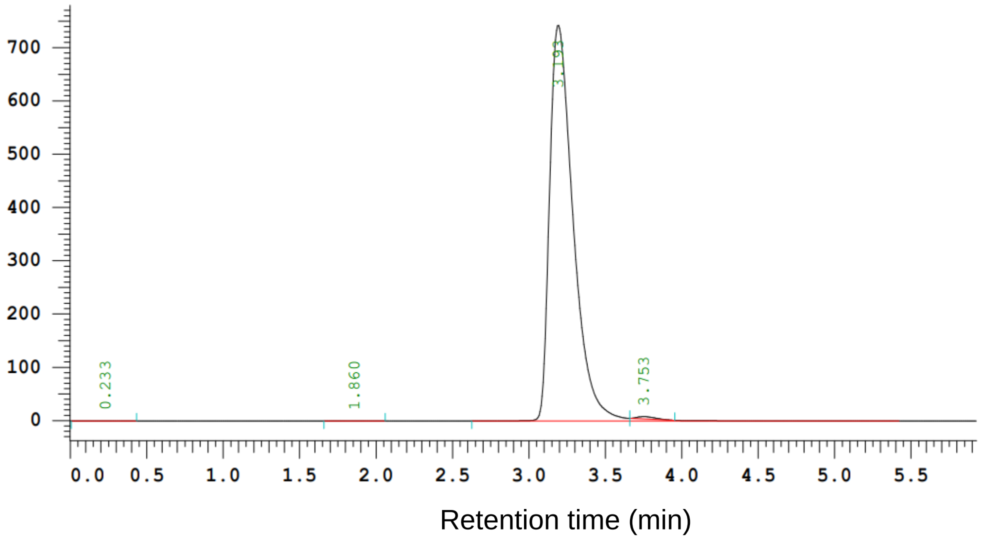

700
600
500
400
300
200
100
0
0.233
1.860
3.193
3.753
0.0
0.5
1.0
1.5
2.0
2.5
3.0
3.5
4.0
4.5
5.0
5.5
Retention time (min)
